# Mononuclear phagocyte system-related multi-omics features yield head and neck squamous cell carcinoma subtypes with distinct overall survival, drug, and immunotherapy responses

**DOI:** 10.1007/s00432-023-05512-5

**Published:** 2024-01-27

**Authors:** Cong Zhang, Jielian Deng, Kangjie Li, Guichuan Lai, Hui Liu, Yuan Zhang, Biao Xie, Xiaoni Zhong

**Affiliations:** Department of Epidemiology and Health Statistics, School of Public Health, Chongqing Medical University, Yixue Road, Chongqing, 400016 China

**Keywords:** Head and neck squamous carcinoma, Mononuclear phagocyte system, Machine learning, Prognosis, Immunotherapy, Drug response

## Abstract

**Background:**

Recent research reported that mononuclear phagocyte system (MPS) can contribute to immune defense but the classification of head and neck squamous cell carcinoma (HNSCC) patients based on MPS-related multi-omics features using machine learning lacked.

**Methods:**

In this study, we obtain marker genes for MPS through differential analysis at the single-cell level and utilize “similarity network fusion” and “MoCluster” algorithms to cluster patients’ multi-omics features. Subsequently, based on the corresponding clinical information, we investigate the prognosis, drugs, immunotherapy, and biological differences between the subtypes. A total of 848 patients have been included in this study, and the results obtained from the training set can be verified by two independent validation sets using “the nearest template prediction”.

**Results:**

We identified two subtypes of HNSCC based on MPS-related multi-omics features, with CS2 exhibiting better predictive prognosis and drug response. CS2 represented better xenobiotic metabolism and higher levels of T and B cell infiltration, while the biological functions of CS1 were mainly enriched in coagulation function, extracellular matrix, and the JAK-STAT signaling pathway. Furthermore, we established a novel and stable classifier called “getMPsub” to classify HNSCC patients, demonstrating good consistency in the same training set. External validation sets classified by “getMPsub” also illustrated similar differences between the two subtypes.

**Conclusions:**

Our study identified two HNSCC subtypes by machine learning and explored their biological difference. Notably, we constructed a robust classifier that presented an excellent classifying prediction, providing new insight into the precision medicine of HNSCC.

**Supplementary Information:**

The online version contains supplementary material available at 10.1007/s00432-023-05512-5.

## Introduction

Head and neck cancer (HNSC) is the sixth most common malignant tumor in the world, with head and neck squamous cell carcinoma (HNSCC) accounting for the majority (Sung et al. 2021). Although some progress has been made in the treatment of HNSCC recently, many patients experience significant declines in swallowing or speech, and the 5-year total mortality rate remains at 60% (Murdoch 2007). Immunotherapy has become an emerging cancer treatment modality that regulates the immune system to fight against tumor cells and mitigates resistance to other treatment modalities, thus having a significant impact on the survival and quality of life of cancer patients (Chen and Mellman 2013). Preclinical data indicate that HNSCC is a deeply immunosuppressive disease characterized by abnormal secretion of pro-inflammatory cytokines and dysfunction of immune effector cells (Gavrielatou et al. 2020). Recent clinical practices have shown that the use of monoclonal antibodies to inhibit the PD-1/L1 checkpoint demonstrates good efficacy in the treatment of various cancer types including HNSCC. Combination therapies using checkpoint inhibitors with radiotherapy and/or chemotherapy, cytokine-based and/or adoptive T-cell therapies have also presented some effectiveness (Wallis et al. 2015). However, only a small number of HNSCC patients indeed have benefited from the widely used immunotherapy strategies in clinical practice (Seiwert et al. 2016), so it is increasingly important to discover new biomarkers for the personalized treatment of patients.

The mononuclear phagocyte system (MPS) is an important component of the body’s immune defense (Zhang and Zhang 2020; Ren et al. 2021) and the primary executor of nanoparticle clearance. Previous researches have shown that MPS is the first and foremost significant obstacle blocking drug carriers to target sites after entering the body, especially in terms of clearing the majority of circulating nanomaterials (Lu et al. 2023). MPS, consisting of monocytes, macrophages, and dendritic cells (DCs), play a role in innate immunity through pathogen sensing and phagocytosis and serve as a cellular component in adaptive immunity by presenting antigens to T cells (Geissmann et al. 2010). Monocytes represent immune effector cells, equipped with chemokine receptors and adhesion receptors, producing inflammatory cytokines, phagocytosing cells, and toxic molecules (Geissmann et al. 2010). They can differentiate into inflammatory DCs or macrophages during inflammation but may be less efficient in the steady state (Auffray et al. 2009). Macrophages are phagocytic cells that can eliminate malignant cells by phagocytosis or by producing soluble factors to induce tumor cell apoptosis. In addition to their direct cytotoxic abilities, macrophages play an important role in regulating the progression of tumors through mechanisms such as angiogenesis, fibrosis, and immune surveillance (Long and Beatty 2013). The secretion products of pDC which is a subtype of DC have immunogenic and tolerogenic functions in tumor immunity (Mitchell et al. 2018; Koucký et al. 2019).

MPS is a part of the tumor immune microenvironment. Previous studies have shown that the proportion of MPS in HNSCC patients varies individually and is usually associated with patient survival and other phenotypes (Balm et al. 1982, 1984a, b). However, in the exploration of biomarkers for HNSCC, previous research has not yet focused on this important component of the immune system. Instead, they have paid more attention to biomarkers associated with several hot topics, such as PDL-1 expression (Dong et al. 2002; Freeman et al. 2000; Topalian et al. 2012), tumor mutational burden/neo-antigens (Charoentong et al. 2017), interferon-γ gene signature (Woo et al. 2015), and tumor microenvironment (Ager and May 2015). There seems to be no previous study using MPS to search for biomarkers and to accurately classify HNSCC patients based on these biomarkers, to achieve further precision treatment. Therefore, differentiated patient classification based on MPS biomarkers is feasible and can provide some references for future personalized treatment of HNSCC.

Based on biomarkers of MPS obtained from single-cell sequencing analysis, this study aimed to recognize HNSCC subtypes with distinct overall survival, drug, and immunotherapy responses. Notably, we can not only consider the impact of gene expression, but also the factor including gene methylation and mutation to have a more comprehensive analysis while classifying HNSCC patients. In addition, to make our research results potentially useful in practice, we constructed a robust classifier based on genes with specific expression in subtypes, which can classify patients even with only gene expression data and had a certain degree of accuracy. The classifier now has been uploaded to GitHub (https://github.com/CQMUZC/getMPsub).

## Materials and methods

### Data source

A single-cell RNA sequencing (scRNA-seq) profile, GSE195832, was obtained from the GEO database (https://www.ncbi.nlm.nih.gov/geo). Considering the aim to explore the marker genes from tumor-infiltrating MPS, four raw scRNA-seq samples, GSM5851565, GSM5851567, GSM5851569, and GSM5851571, were included in our analysis. TCGA HNSCC multi-omics feature regarding RNA-seq, methylation, and mutation were attained as the training dataset from UCSC Xena (https://xenabrowser.net/) to form the specific HNSCC subtype. GSE41613 and GSE65858 were applied as the validation datasets to confirm whether the subtypes had strong universality and to verify certain effectiveness of the classifier.

### Single-cell analysis

We first paid attention to the global quality of the mixed data including Mitochondrial percentage, the count of expression in samples per gene, and the count of gene expression per sample. Only cells with a mitochondrial percentage below 25% and gene expression numbers between 600 and 7500 will be included in the analysis. In addition, only genes expressed in more than 1000 cells will be considered. Given the batch effect from different samples, we employed “harmony” reduction to diminish the system error (Korsunsky et al. 2019; Azizi et al. 2018). We performed standardization and normalization on the preprocessed expression matrix so that gene expression levels of each cell can be compared and further analyzed. Principal component analysis (PCA) was adopted to reduce the data noise and uniform manifold approximation and projection (UMAP) was utilized to further depict cell clusters clearly. Given the results of “clustree” and the specific biological targets (Zappia et al. 2018), cells were clustered by integrated results from graph-based clustering and shared nearest-neighbor clustering. Then we annotated cell clusters and verified each other by two methods, which used the R package “SingleR” based on “celldex” to auto-annotate cells and some marker genes to manually annotate. According to the results, R function “FindAllMarkers” was applied to identify differentially expressed genes (DEGs) between each cell cluster and others. Through all the above analysis, we determined MPS and detected their marker genes to deepen our understanding. To confirm the identity of these marker genes, Enrichr (https://maayanlab.cloud/Enrichr/) was adopted to identify which cells will be enriched by these markers. And KEGG pathway enrichment analysis was employed to represent their biological function.

### Clustering analysis in multi-omics features

MPS-related muti-omics were identified by the intersection of gene variables and markers from the single-cell analysis. R package “MOVICS” was utilized to characterize the HNSCC subtypes by unsupervised clustering (Lu et al. 2021). In the beginning, R function “getElites” filtered features that met some stringent requirements, in which “Cox” was used for RNA-seq and methylation while “freq” was for binary omics data. Then the optimal number of clusters was acquired referring to Cluster Prediction Index (CPI) and Gaps-statistics (Chalise and Fridley 2017). Considering the silhouette score and the final survival difference, SNF (Wang et al. 2014) and MoCluster (Meng et al. 2016) performed the consensus clustering and recognized the HNSCC subtypes. The overall nominal *P*-value was calculated by log-rank test and Kaplan–Meier (KM) Curve was printed to show the HNSCC subtypes’ survival difference. Finally, based on the specific expression genes of two subtypes, we aimed to develop a classifier that can predict the subtypes of other HNSCC patients using only RNA-seq.

### Drug sensitivity

Genomics of Drug Sensitivity in Cancer (GDSC) are a public database containing cancer cells’ drug sensitivity and molecular markers corresponding to the applied drugs. Among patient subtypes, we considered the differences in four small molecule compounds, Paclitaxel, 5-Fluorouracil, Erlotinib, and Pazopanib. We tested the differential drug response of two clusters to nanomedicines, because Abraxane, a nano-subtype of paclitaxel, was included in the GDSC drug database. 5-Fluorouracil, as the main clinical treatment for HNSCC, was utilized to test whether the subtype had a distinct drug sensitivity for conventional treatment methods. Erlotinib and Pazopanib were utilized to test whether there was a response to specific targets, EGFR and CSF1R. Given the effect of these drugs used in combination with radiotherapy, we subsequently test the differential drug response of patients who had records of radiation therapy in two clusters. Independent-samples *t*-test was performed to determine the differences in two clusters. Kruskal–Wallis rank sum test was performed for multiple subtypes.

### Tumor immune microenvironment

“CIBERSORT” algorithm (https://cibersort.stanford.edu/) evaluated the infiltration degree of 22 immune cells between the two subtypes. EPIC can analyze the expression matrix to determine the infiltration proportions of eight types of immune cells, including B cells, cancer-associated fibroblasts (CAFs), CD4^+^ T cells, CD8^+^ T cells, endothelial cells, macrophages, and NK cells, which were all important components of the immune microenvironment. TIMER utilized a deconvolution algorithm to infer the abundance of tumor-infiltrating immune cells from gene expression profiles. The immune-infiltrating situation of different subtypes can be corroborated given the results of the above algorithms. Additionally, “TIDE” algorithm was employed to evaluate the potential clinical efficacy of immunotherapy in different subtypes and reflected the underlying ability of tumors to escape the immune system. The TIDE score evaluated the potential response to immune checkpoint therapy, with a higher score indicating a poorer response to this treatment and may require alternative therapies. The Exclusion score evaluated the degree of infiltration of immune-suppressive cells in the tumor microenvironment. The higher score indicated a more severe infiltration of immune-suppressive cells and a poorer response to immune checkpoint therapy. The Dysfunction score evaluated the functional state of immune cells in the tumor microenvironment. The higher score demonstrated that the function of immune cells was suppressed in the tumor microenvironment, leading to a poorer response to immune checkpoint therapy. Besides, the higher MSI score corresponded to a higher level of immune cell infiltration and stronger immune response.

### MPS-related analysis

The abundance of MPS including macrophages, DCs, and monocytes was calculated by “IOBR” (Zeng et al. 2021). Some targets, CSF1R, TLR8, EFGR, CXCR4, ABCA1, MGFE8, CD47, and CX3CL1, related to tumor-associated macrophagocytes (TAMs), immune therapy in HNSCC, and efferocytosis were detected to explore whether subtypes expressed differently.

### Functional analysis

GO was a database established by the Gene Ontology Consortium, aimed at describing gene and protein function. Through GO enrichment analysis, this study can roughly annotate genes and classify them according to biological processes, molecular function, and cellular component. KEGG was a database that systematically analyzed gene function, links genomic information and functional information. The Hallmark gene set was a collection of genes developed jointly by the Human Cell Atlas and the Genomics Institute of the Novartis Research Foundation. The gene set was generated from cell-type-specific genomic expression data and contained gene markers for multiple tissue and cell types, which can be utilized to identify and analyze differences between different cell types or states. In this study, GSEA enrichment analysis was performed using the three different reference gene sets of GO, KEGG, and Hallmark to validate the specific biological differences exhibited by two subtypes. Pathways enriched in two or more reference gene sets were considered to represent unique biological functions specific to the subtype.

### Integrated metabolism analysis

In addition to representing the immune microenvironment of subtypes, the R package “IOBR” had been used to evaluate metabolic differences between two subtypes. This study assessed the metabolic levels of subtypes from three perspectives: metabolism, fatty acid metabolism, and cholesterol metabolism, aiming to explore the relevant differences.

### The integrated analysis of copy number variation

Considering subtype-specific mutation might be promising as therapeutic target, this study compared the mutational frequency among different subtypes. R package “MOVICS” offered two functions to measure genomic alterations potentially affecting immunotherapy, namely the quantification of total mutation burden (TMB) and fraction genome altered (FGA). In addition, the function “compFGA” calculated and compared not only FGA but also computed specific gain (FGG) or loss (FGL) per sample within each subtype. To measure the consistency of current subtypes with other pre-existing classifications, “MOVICS” offered the function “compAgree” to generate alluvial diagram, visualizing the consistency of two evaluation phenotypes with the current subtype as a reference.

### Statistical analysis

All the data processing and analyses were executed in R software (Version 4.2.2). *t*-Test and Wilcoxon test were utilized to compare the differences between quantitative variables while Chi-square test was employed in qualitative variables. Spearman correlation test was utilized to explore the relationships between variables. The Kappa coefficient was utilized to measure the level of agreement between classifier results and actual classifications. A KAPPA value below 0.4 indicated poor agreement, 0.4–0.6 indicated moderate agreement, 0.6–0.8 indicated good agreement, and above 0.8 indicated excellent agreement. *P* < 0.05 was considered statistically significant in the whole process.

## Results

### Data processing

**Fig. 1:**
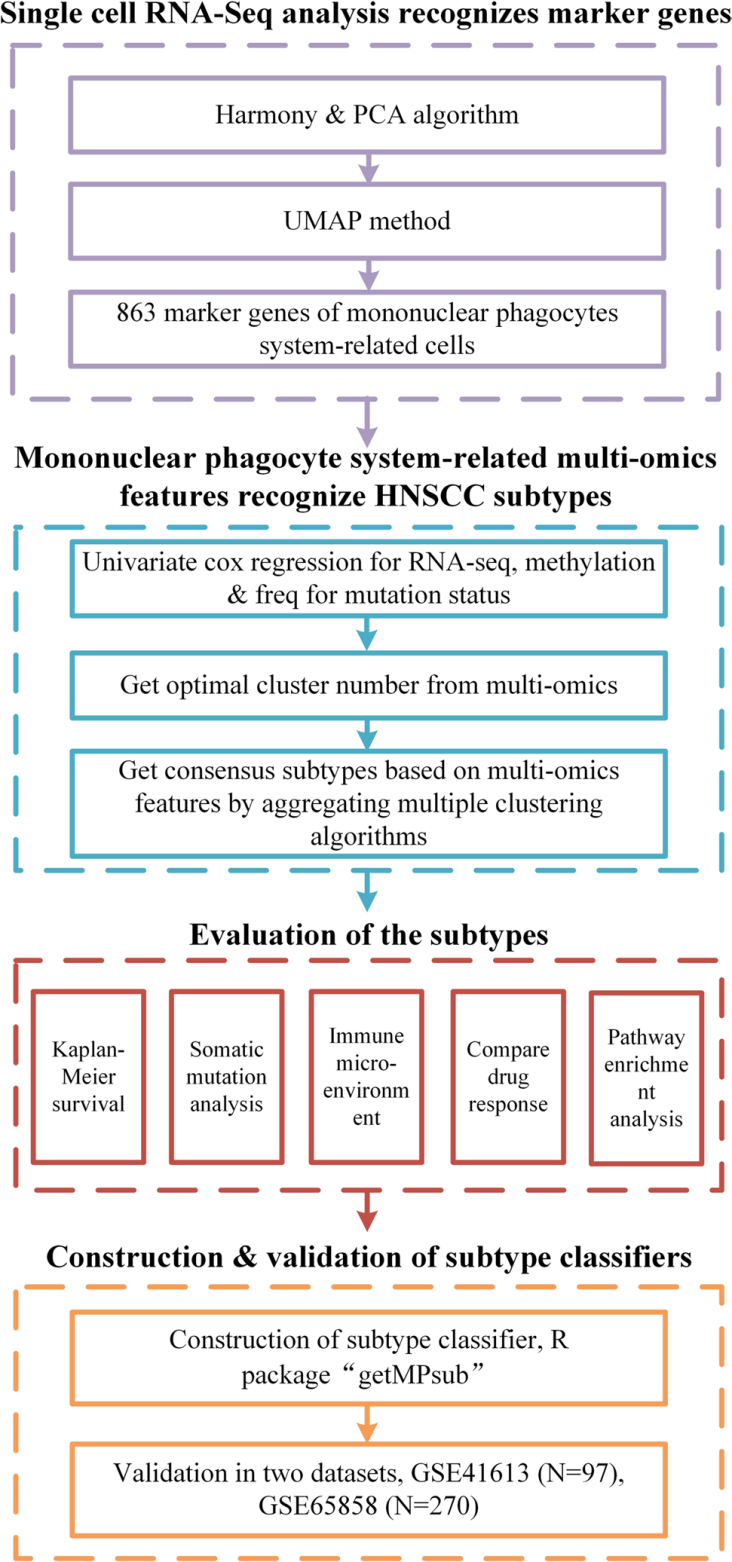
The flowchart of the present study

The main process of this study, including the analysis involved, is specifically shown in Fig. 1. Considering integrity and commonality, 848 HNSCC patients were included as the working data when patients containing missing information were excluded. Among them, 481 TCGA-HNSCC patients were included to train the classifier while 270 HNSCC samples in GSE65858 and 97 HNSCC samples in GSE41613 were enrolled, respectively, as two validation datasets. The basic characteristics including the origin, form, and some clinical characteristics of data per dataset is displayed in Table 1.

**Table 1:** The summary characteristics of the included samples in this study

Datasets		
TCGA-HNSC		
Sources	Data types	Samples
TCGA	Gene expression RNAseq	481
	Range	Mean
Age	20–90	61.54
	No	%
Age		
19–39	16	3.33
40–49	51	10.60
50–59	115	23.91
60–88	299	62.16
Gender		
Female	131	27.23
Male	350	72.77
Clinical stage		
Stage I	18	3.74
Stage II	93	19.34
Stage III	102	21.20
Stage IVa	253	52.60
Stage IVb	9	1.87
Stage IVc	6	1.25
Radiation therapy		
Yes	245	50.94
No	132	27.44
Not reported	104	21.62

GSE65858		
Sources	Data types	Samples
GEO	gene expression RNAseq	270
	Range	Mean
Age	35–87	60.12
	No	%
Age		
19–39	2	0.74
40–49	39	14.44
50–59	100	37.04
60–88	129	47.78
Gender		
Female	47	17.40
Male	223	82.60
Clinical stage		
Stage I	18	6.67
Stage II	37	13.70
Stage III	37	13.70
Stage IVa	155	57.41
Stage IVb	16	5.93
Stage IVc	7	2.59
Treatment		
Uni-modality	78	28.89
Multi-modality	189	70.00
Palliative	3	1.11

GSE41613		
Sources	Data types	Samples
GEO	gene expression RNAseq	97
	No	%
Age		
19–39	6	6.19
40–49	16	16.49
50–59	28	28.87
60–88	47	48.45
Gender		
Female	31	31.96
Male	66	68.04
Clinical stage		
Stage I/II	41	42.27
Stage III/IV	56	57.73
Treatment		
Uni-modality	43	44.33
Multi-modality	53	54.64
Not reported	1	1.03

GSE195832		
Sources	Data types	Samples
GEO	Single-cell RNAseq	Not applicable

### Single-cell analysis recognized marker genes

**Fig. 2:**
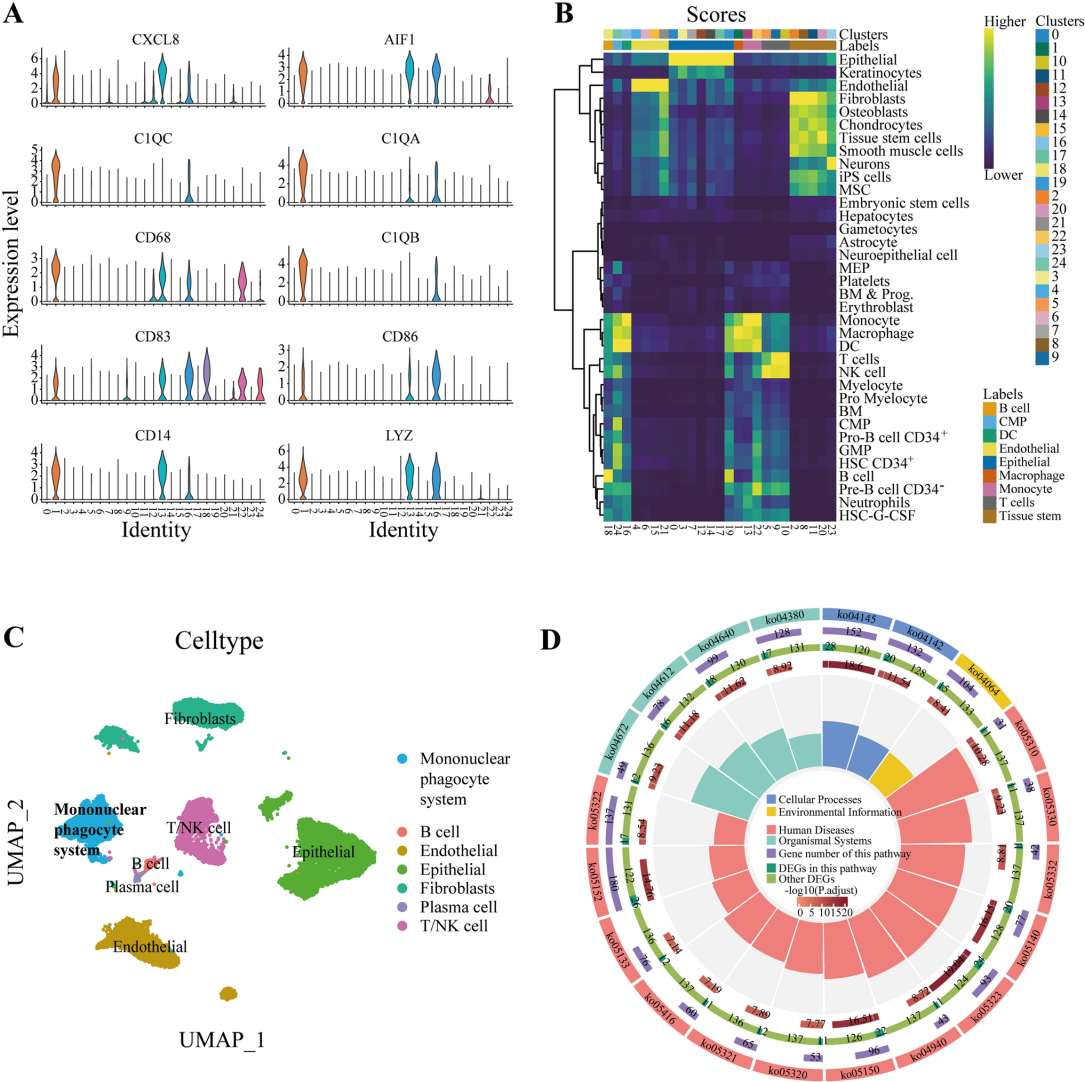
Single-cell analysis recognized marker genes. **A** The expression level of MPS specific genes in 25 cell clusters. **B** The heatmap of expression level in 25 clusters. **C** Annotation results under the UMAP reduction. **D** KEGG analysis characterized phagocytosis-related biological functions

Through diminishing the batch effort, four raw scRNA-seq samples presented a uniform and random distribution under the UMAP reduction (Figure S1A). 25 cell clusters were gathered, of which a smaller number responded to more cells (Figure S1B). Some genes, CXCL8, AIF1, C1QC, C1QA, CD68, C1QB, CD83, CD86, CD14, and LYZ, that have been confirmed to be specifically expressed in MPS are used as manually annotated marker genes to view their expression in 25 cell clusters. Cluster 1, 13, and 16 were the cell populations with high expression of these genes (Fig. 2A). Besides, other cells were identified for gene expression using corresponding marker genes. Based on their expression level, each cell cluster was ultimately annotated as a specific cell population, in which cell clusters with high or no expression of multiple marker genes were defined as “unknown”. Besides, R package “SingleR” recognized certain cells referring to “celldex”. The corresponding heatmap (Fig. 2B) depicted the expression level of various cells in 25 clusters. Finally, we presented the annotation results under the UMAP reduction, showing their specific clusters (Fig. 2C). Almost cell clusters had the same definition except for “unknown”, and “Fibroblasts”, which heatmap resulted by “SingleR” also had a similar expression level compared with manual annotation. For instance, the heatmap indicated Fibroblasts reference expressed highly in cluster 2, 20, and 11, defined as “tissue stem cells” by SingleR, which is consistent with our manual annotation. Notably, clusters 1, 13, and 16 were manually annotated as mononuclear phagocytes including monocytes, macrophages, and DCs, that is, manual annotation and SingleR had the same determination regarding the targeted cells. 863 marker genes of mononuclear phagocytes were found by R function “FindAllMarkers” (Table S3) and were corroborated enriching in macrophages, monocytes, and DCs by Enrichr (Table S1). KEGG analysis presented that the pathway enriching the most genes was “Phagosome”, indicating our marker genes indeed characterized phagocytosis-related biological functions (Fig. 2D).

### Clustering analysis recognized HNSC subtypes

**Fig. 3:**
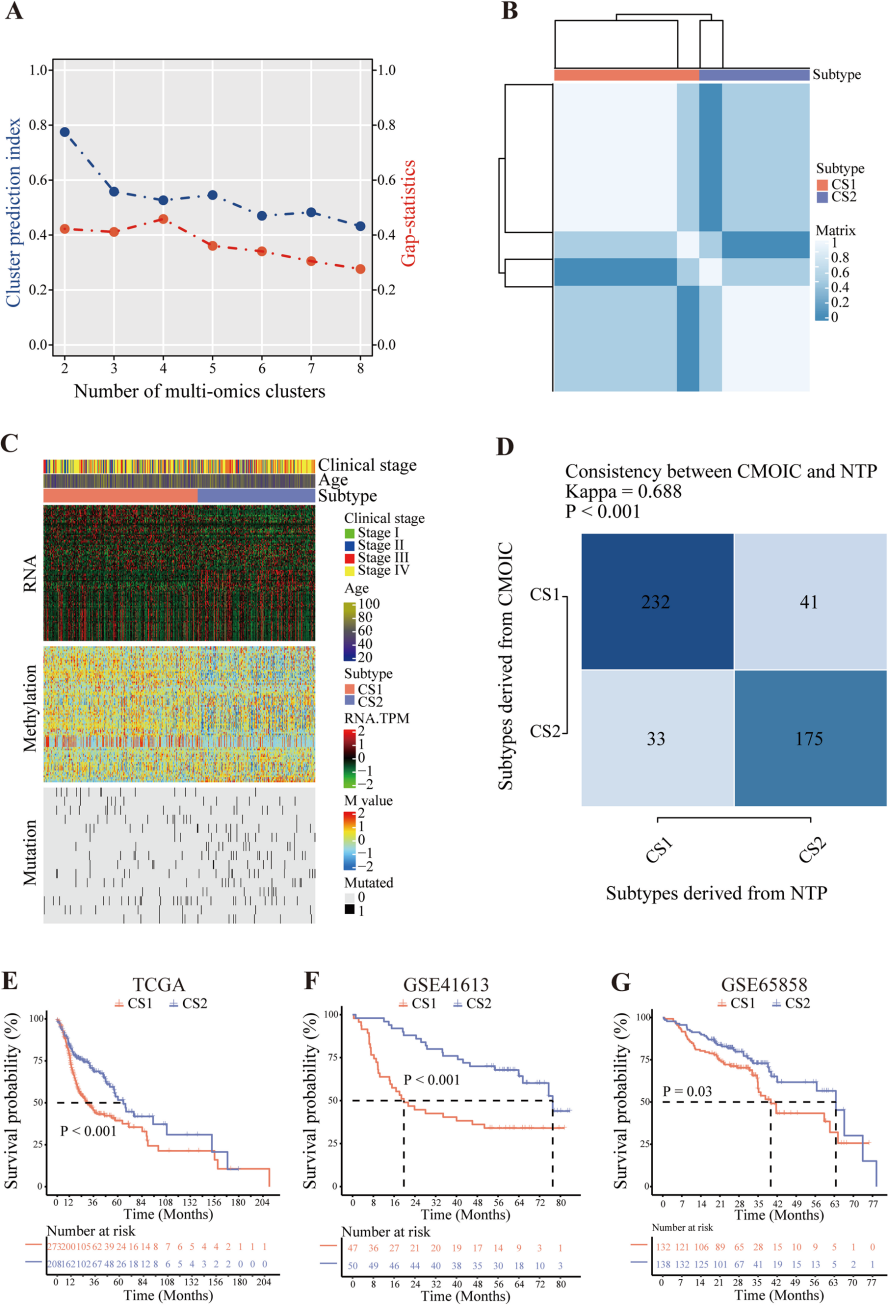
Clustering analysis recognized HNSC subtypes. **A** The number of multi-omics clusters. **B** A stable clustering result by applying hierarchical clustering. **C** The heatmap of overall clustering process. **D** The KAPPA value of the classifier. **E** The KM curve of TCGA cohort. **F** The KM curve of GSE41613. **G** The KM curve of GSE65858

Given the integrated results by the CPI and Gap-statistics, the imputed optimal cluster was 2 (Fig. 3A). The consensus heatmap depicted robust pairwise similarities for two subtypes and the details of how it got a stable clustering result by applying hierarchical clustering (Fig. 3B). The genome-wide heatmap was utilized to reveal information about how the samples cluster together and provide insights into potential sample biases or other artifacts (Fig. 3C). Additionally, it offered the difference in some phenotypes such as age and clinical stage between the two subtypes. According to subtype-specific biomarkers (Table S2), we established an HNSC classifier using nearest template prediction (NTP) to predict the possible subtypes of each sample. The Kappa values, evaluating the performance of the HNSC subtypes classifier, represented a good consistency in predicting the subtype for HNSC samples by the comparison of the actual subtype and the predicted type in the training dataset (Fig. 3D). The consensus clustering resulted in the HNSC subtypes with distinct survival differences in the training dataset, in which samples in cluster 2 were more likely to have a better prognosis (Fig. 3E). GSE41613 and GSE65858 were considered the validation dataset to confirm the effectiveness of the HNSC classifier. The KM curve indicated that cluster 2 identified by the classifier had a longer overall survival time (Fig. 3F, G). Notably, for the convenience of future research, we have packaged the classifier using the NTP algorithm into an R package called “getMPsub” and uploaded it to GitHub for easy accessibility.

### Drug sensitivity

**Fig. 4:**
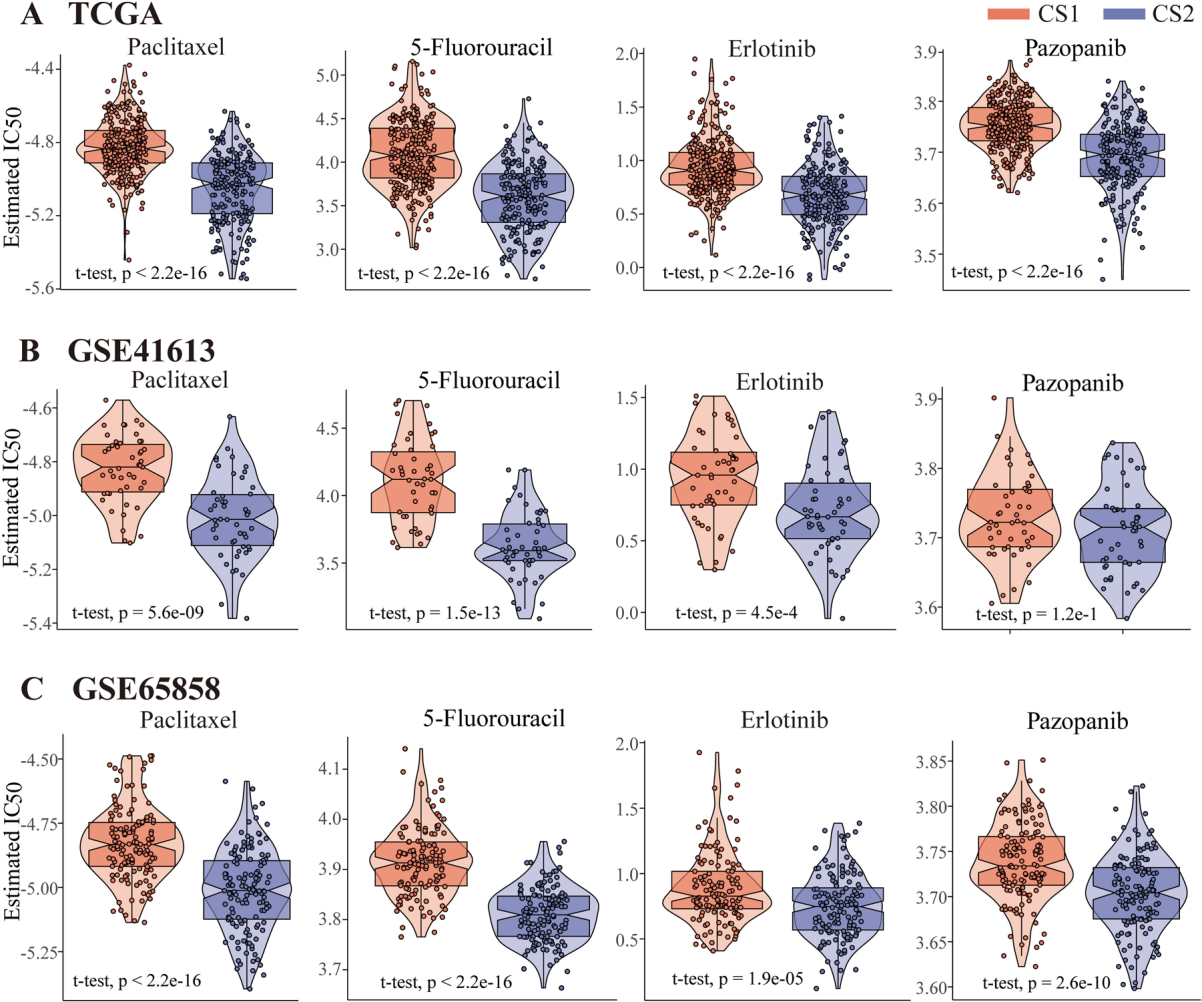
The comparison of two subtypes’ drug sensitivity. **A** The estimated IC50 of paclitaxel, 5-fluorouracil, erlotinib, and pazopanib between two subtypes in TCGA cohort. **B** The estimated IC50 of paclitaxel, 5-fluorouracil, erlotinib, and pazopanib between two subtypes in GSE41613. **C** The estimated IC50 of paclitaxel, 5-fluorouracil, erlotinib, and pazopanib between two subtypes in GSE65858

The *t*-test showed significant differences in sensitivity to four small molecule compounds between two subtypes, with cluster 2 exhibiting lower IC50 values, indicating greater sensitivity to these drugs. Both in the TCGA training set and in the two validation sets, the drug sensitivity of the two subtypes represented similar differences, which also verified the robustness of our classifying strategy (Fig. 4A–C). According to the NCCN (National Comprehensive Cancer Network, https://www.nccn.org/) guidelines for head and neck cancer treatment, these drugs were used in combination with radiotherapy in first-line treatment and in previous studies. Therefore, we do corresponding drug sensitivity tests on patients from the TCGA cohort who had records of radiation therapy. Further testing revealed that CS1 exhibited similar drug sensitivity regardless of its history of radiation therapy while CS2 patients, after undergoing radiation therapy, were more sensitive to 5-FU and Pazopanib compared to those without radiation therapy. (Figure S13).

### Immune cell infiltration analysis

**Fig. 5:**
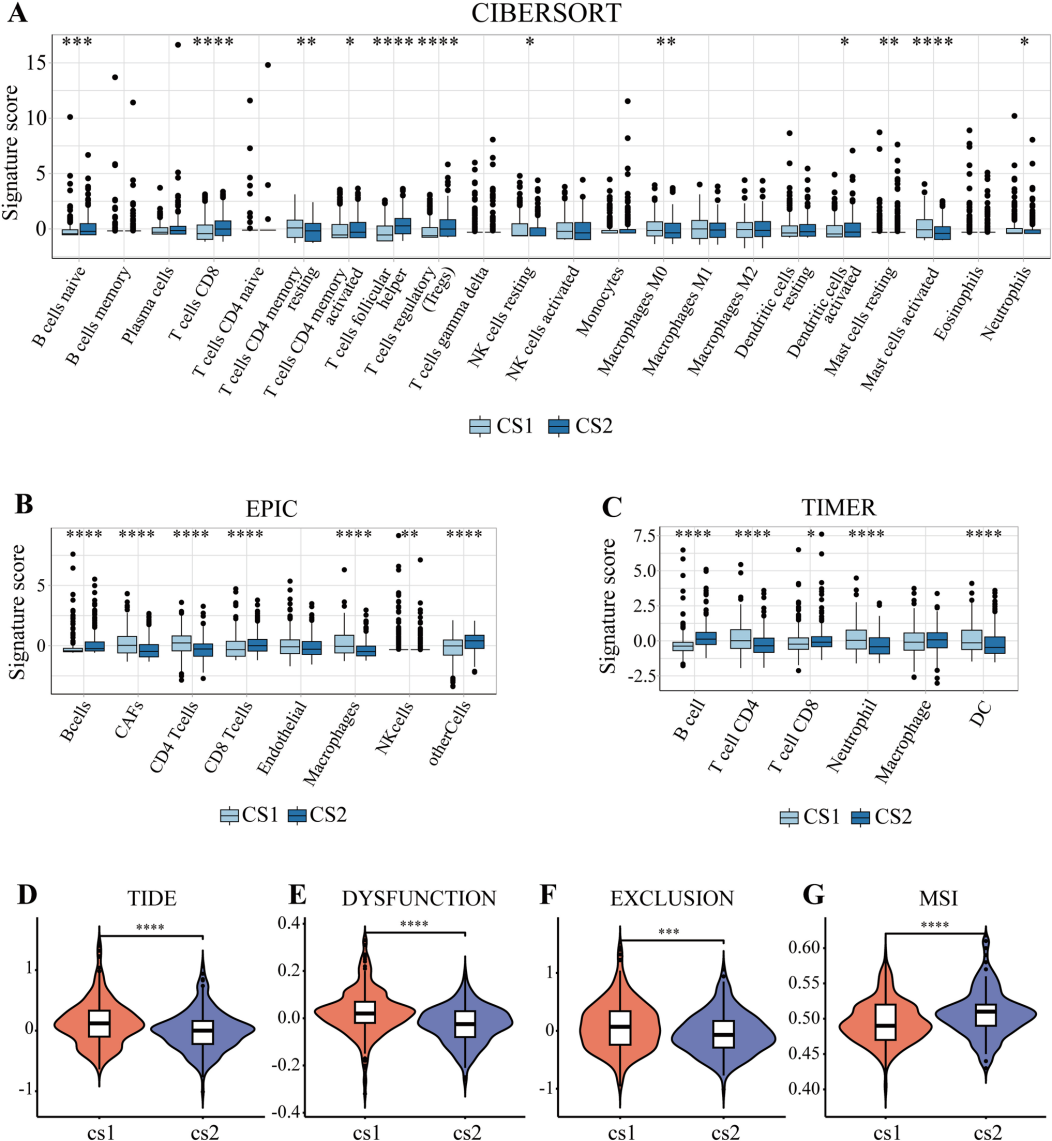
The comparison of two subtypes’ immune cell infiltration. **A** Differences in 22 immune cells between two subtypes by algorithm CIBERSORT. **B** Differences in eight immune cells between two subtypes by algorithm EPIC. **C** Differences in six immune cells between two subtypes by algorithm TIMER. **D** The TIDE score of two subtypes. **E** The Dysfunction score of two subtypes. **F** The Exclusion score of two subtypes. **G** The MSI score of two subtypes (ns/empty space, no significance, *p < 0.05, **p < 0.01, ***p < 0.001, and ****p < 0.0001)

Tumor immune cell infiltration in recent researches was widely considered a crucial feature of the TME. The boxplot illustrated the different abundance of immune cells between the TME of two HNSC subtypes via three algorithms, CIBERSORT, EPIC and TIMER. In the CIBERSORT results, cluster 2 in three datasets tended to represent a higher degree of immune infiltration in almost all 22 immune cells, except in M1 macrophages, M2 macrophages, resting DCs, Neutrophils, activated Mast cells, resting memory CD4 T cells, and memory B cells (Fig. 5A, Figs. S7, S8). Similar results were acquired by the EPIC and TIMER algorithm, showing that the cluster 2 had a higher level of immune infiltration in main immune cell, CD8 T cell, and a lower level of some cells related to MPS (Fig. 5B, C, Figs. S7B, C, S8B, C). The difference in the degree of specific cell infiltration might be caused by the number of immune cells and different statistical methods used. Above the results, cluster 2 performed better. As a supplement, cluster 2 had a lower level of TIDE score, Exclusion score, but a higher score of MSI, indicating a better performance of the potential clinical efficacy of immunotherapy (Fig. 5D–G, Figs. S7D–G, S8D–G).

### MPS-related analysis

**Fig. 6:**
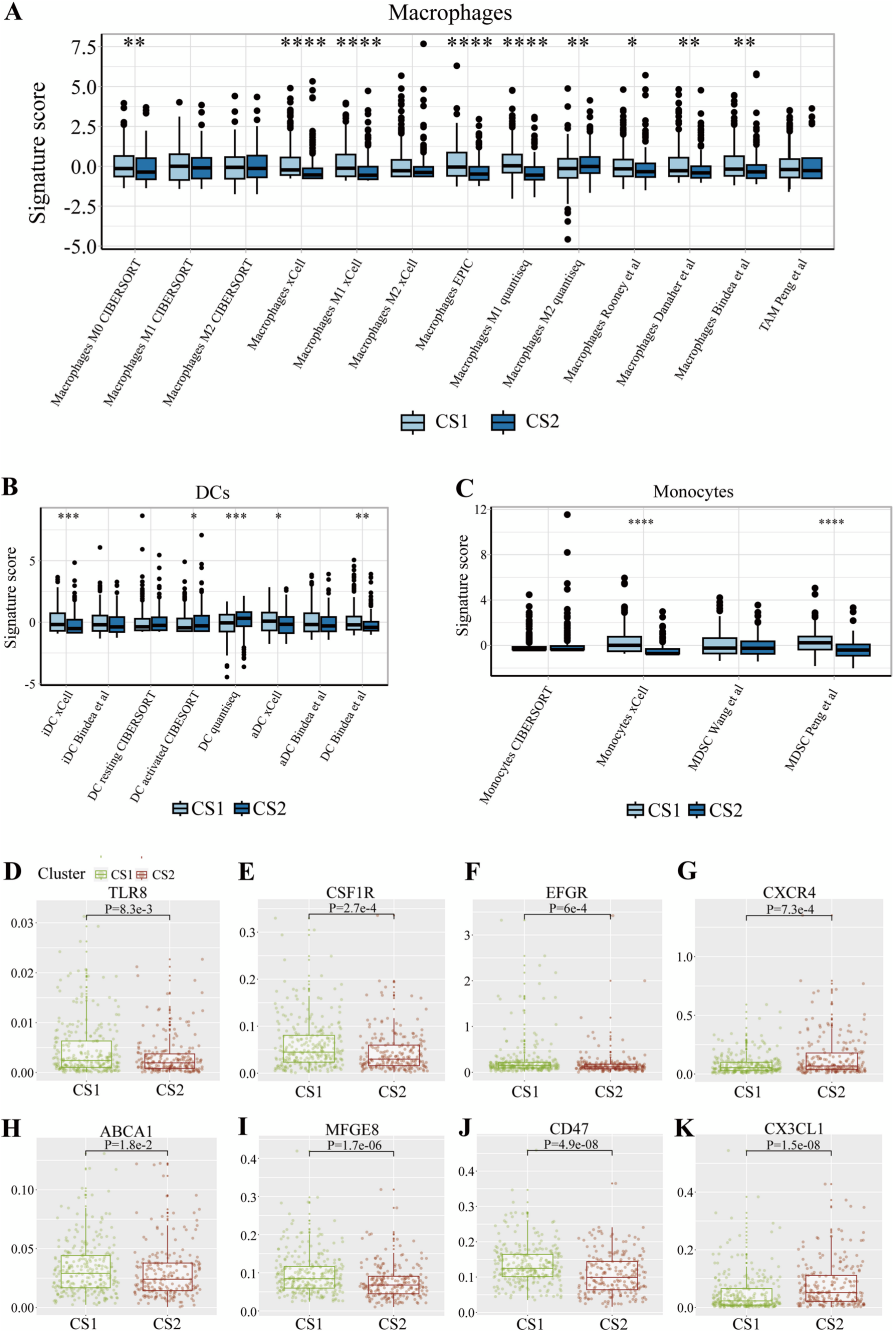
MPS-related analysis. **A** The specific proportion of macrophages between two subtypes. **B** The specific proportion of DCs between two subtypes. **C** The specific proportion of monocytes between two subtypes. **D-K** The expression level of TLR8, CSF1R, EGFR, CXCR4, ABCA1, MFGE8, CD47, and CX3CL1, between two subtypes. (ns/empty space, no significance, *p < 0.05, **p < 0.01, ***p < 0.001, and ****p < 0.0001)

We examined the cell proportions of MPS in HNSC subtypes and found that cluster 2 in most gene signature, at a statistical level, had lower scores for macrophages, monocytes, and DCs, indicating that patients in cluster 2 had a lower proportion of MPS cells (Fig. 6A–C, Figs. S9A–C, S10A–C). This study then detected the immune checkpoint targets related to MPS. In the traditional HNSC targets, cluster 2 had a lower expression level of EFGR and a higher expression level of CXCR4 (Fig. 6F, G), with similar trend or similar statistical meaning in two validations (Figs. S9F, G, S10F, G). Considering that MPS included TAMs, we also tested the relevant targets for TAMs. Cluster 2 had a higher expression level of CSF1R and a lower expression level of TLR8 (Fig. 6D, E). Recent studies had also found that MPS more or less participated in efferocytosis, so this study also tested whether there were expression differences in signaling molecules and targets for efferocytosis in HNSC patient subtypes. Finally, the result depicted that CD47, MFGE8, and ABCA1 all had lower expression levels in cluster 2, while CX3CL1 had a higher expression level in cluster 2 (Fig. 6H–K). MFGE8, ABCA1, and CX3CL1 all presented the same results in at least one validation set (Figs. S9H–K, S10H–K).

### Functional analysis

**Fig. 7:**
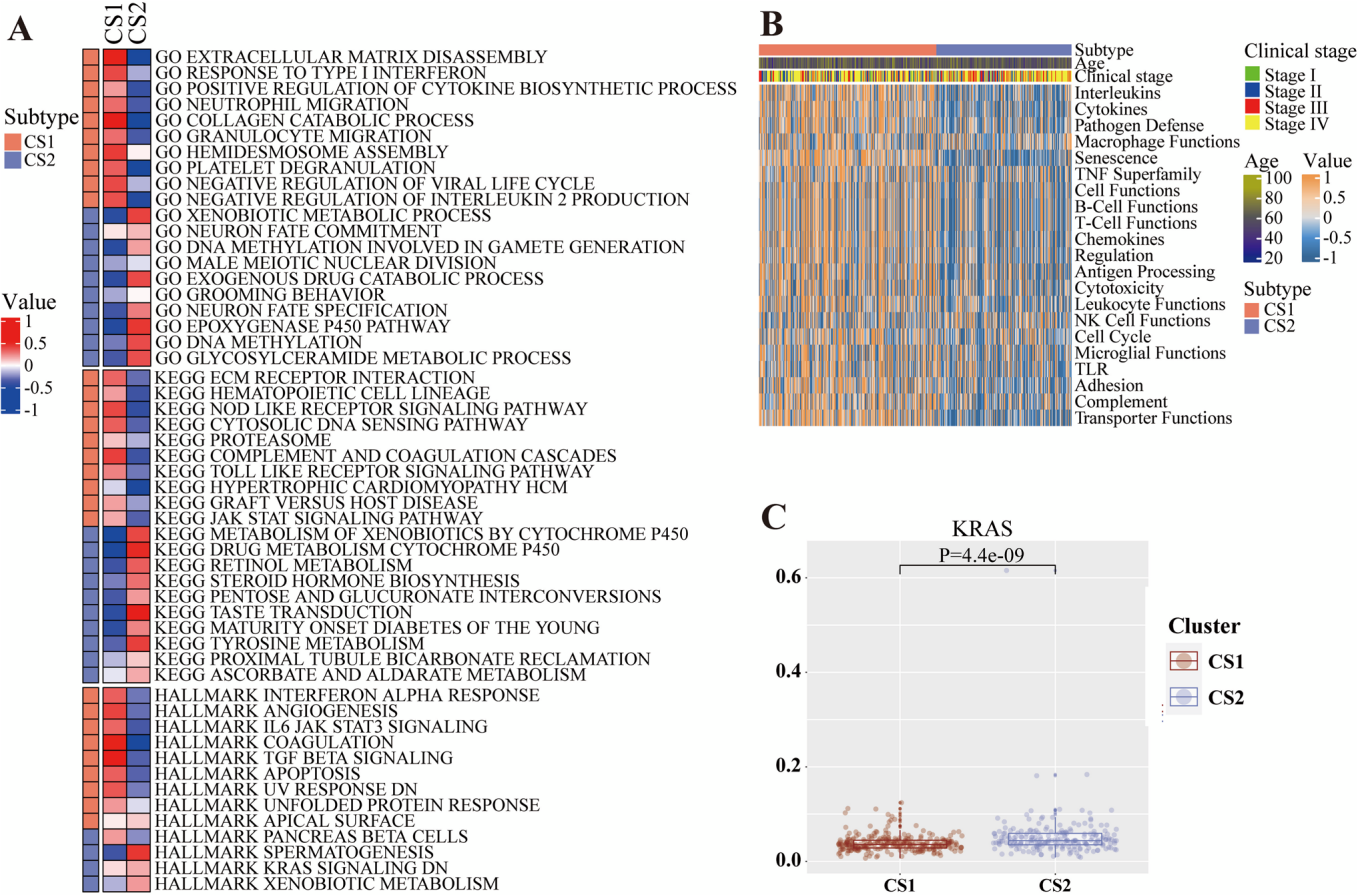
Functional analysis of two subtypes. **A** The pathway enrichment analysis of two subtypes by three algorithms such GO, KEGG, and HALLMARK. **B** The difference of biological function between two subtypes. **C** The expression level of KRAS between two subtypes

Enrichment analysis showed that there were many pathways related to immunotherapy in both clusters, such as the JAK-STAT signaling pathway in cluster 1 and the KRAS signaling pathway in cluster 2. However, pathways enriched in two or more reference sets showed differences in different subtypes. Cluster 1 enriched pathways were related to coagulation function, extracellular matrix (ECM), and JAK-STAT signaling pathway, while cluster 2 enriched pathways were related to xenobiotic metabolism and DNA methylation (Fig. 7A, Figs. S3, S4). Biological functional GSVA analysis using the gene sets built in “MOVICS” represented significant differences between the two subtypes in interleukins, cytokines, pathogen defense, and senescence (Fig. 7B, Figs. S11A, S12A). The KRAS expression levels between the two subtypes in TCGA, GSE65858 were depicted using box plots, indicating significant statistical differences (Fig. 7C, Fig. S12B).

### Metabolism analysis

**Fig. 8:**
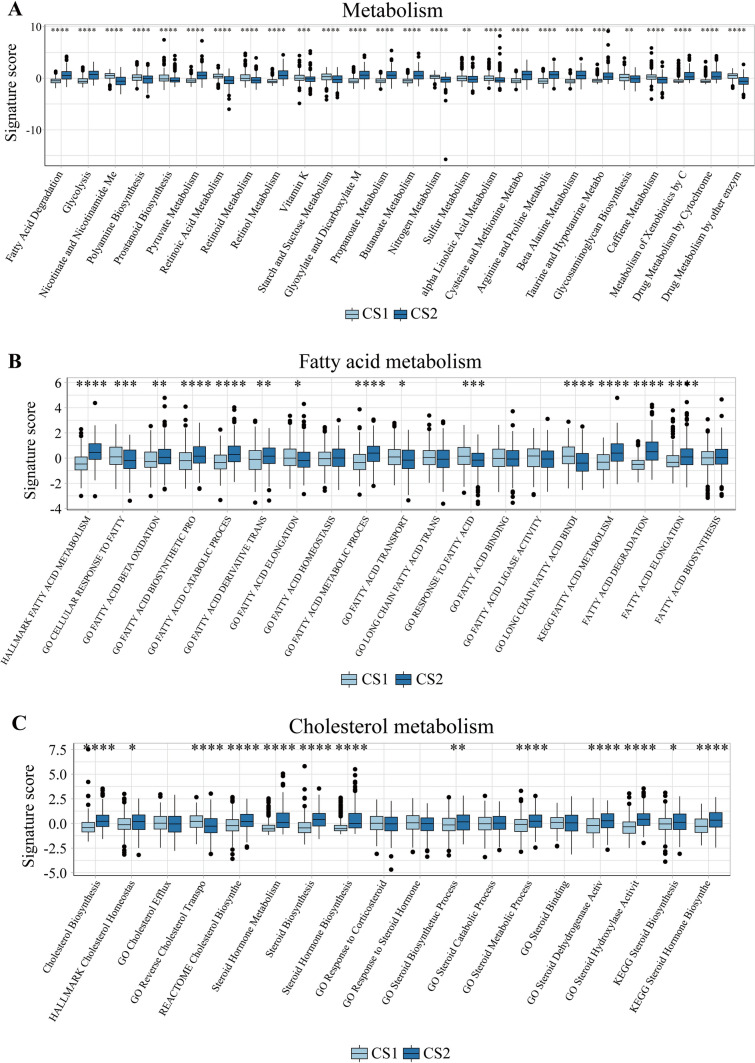
Metabolism analysis of two subtypes. **A** The overall metabolism analysis between two subtypes. **B** The fatty acid metabolism analysis between two subtypes. **C** The cholesterol metabolism analysis between two subtypes. (ns/empty space, no significance, *p < 0.05, **p < 0.01, ***p < 0.001, and ****p < 0.0001)

This study also investigated metabolic differences between subtypes, including overall metabolism, fatty acid metabolism, and cholesterol metabolism. In most cases, cluster 2 had higher metabolic scores, corresponding to better metabolic capabilities. Notably, cluster 2 had a higher level of drug metabolism by cytochrome than cluster 1 while cluster 2 had a relatively lower level of drug metabolism by other enzyme, suggesting the talent difference of drug using (Fig. 8A, Figs. S5A, S6A). The expression level of fatty acid metabolism revealed that cluster was more likely to have a better ability of fatty acid metabolism (Fig. 8B, Figs. S5B, S6B). The level of cholesterol metabolism also had the similar tendency (Fig. 8C, Figs. S5C, S6C).

### The integrated analysis of copy number variation

**Fig. 9:**
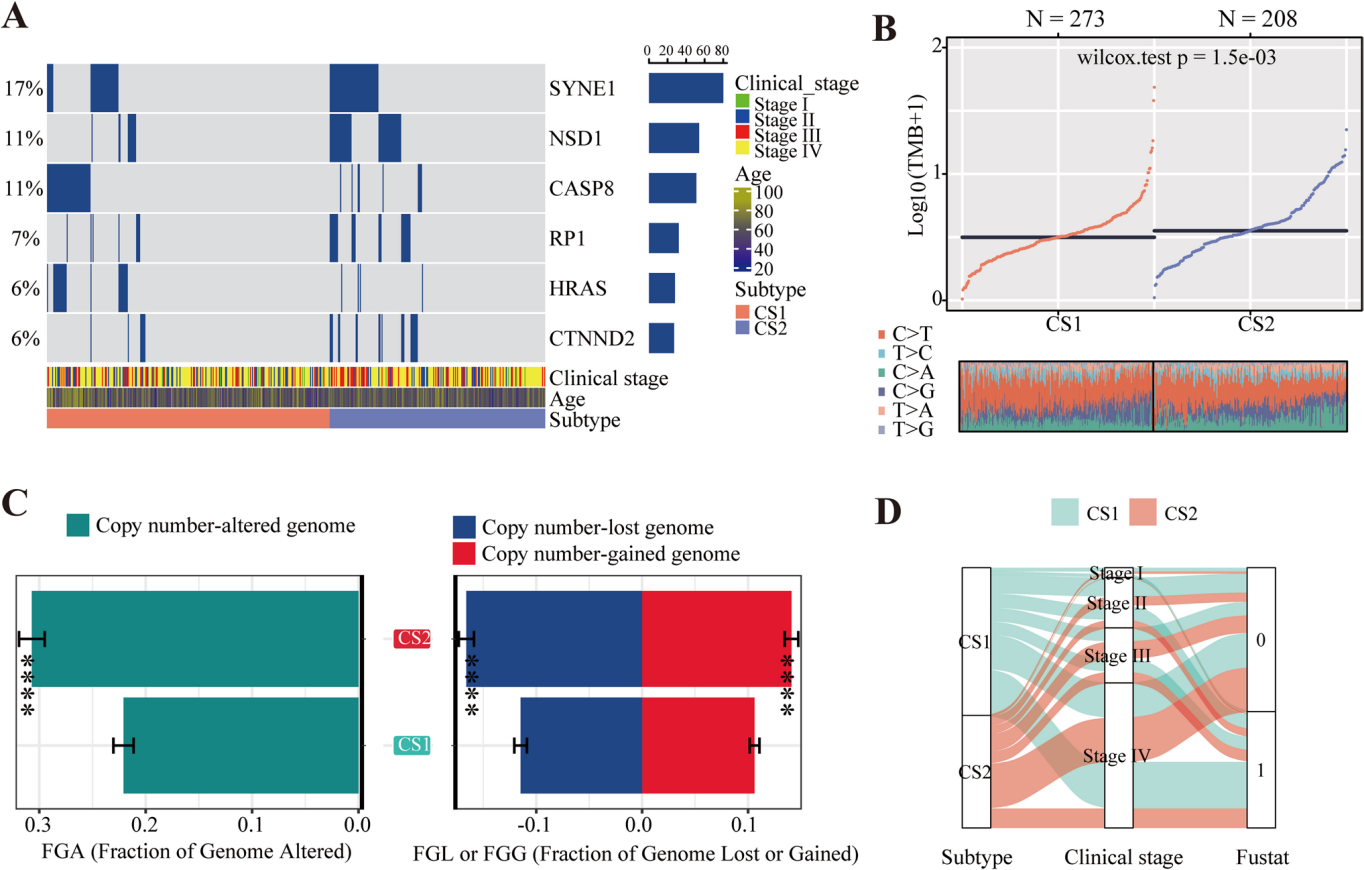
The integrated analysis of copy number variation. **A** The comparison of the mutational frequency between two subtypes. **B** The level of TMB between two subtypes. **C** The comparison of FGA, FGL, and FGG between two subtypes. **D** Consistency between subtypes and other clinical phenotypes. (0 means alive and 1 means death)

Comparing the mutational frequency among different clusters, this study noticed SYNE1, NSD1, CASP8, RP1, HRAS, CTNND2 had a statistical difference (Fig. 9A). Cluster 2 had a higher level of TMB, which is a favorable prognostic factor for cancer (Fig. 9B). Besides, the boxplot (Fig. 9C) demonstrated that cluster 2 had a more copy number-altered genome, whether lost or obtained. The alluvial diagram illustrated the differences in existing phenotypes between different subtypes, which can be seen that Cluster 2 has a higher proportion of survival status in a smaller number of people compared to Cluster 1 (Fig. 9D, Figs. S11D, S12D).

## Discussion

An increasing number of studies had discovered the significant role of MPS in TME, and the regulation of HNSCC progression by MPS had stirred a heated discussion. Nearly all studies aimed at the overall influence of immune system on HNSCC pathobiology, and there seemed to be a lack of the exploration and application of MPS biomarkers for classifying HNSCC patients and verifying, from the statistical perspective based on the clinical data, whether MPS had an impact on the growth process of HNSCC. Therefore, the work of this study is both reasonable and necessary.

This study first detected the MPS marker genes at the single-cell level. By validating the results of manual and automatic annotation, we found that MPS in the single cell RNA seq samples included monocytes, macrophages, and DCs. Comparing the differences between cells, we identified genes that were specifically expressed in MPS. Based on the MPS marker genes, we utilized comprehensive clustering analysis mixing the results of “SNF” and “MoCluster” to divide 481 HNSC patients in TCGA into two subtypes, CS1 and CS2. KM curve and other functional analyses showed that CS2 had a better prognosis, and the two subtypes showed significant differences in multiple aspects such as drug sensitivity, tumor mutations, and enriched pathways, providing a certain differentiation strategy for clinical treatment of HNSCC. In addition, according to the specific expression genes of CS1 and CS2, we constructed a classifier that can perform corresponding stratification with only patient gene expression profile data. The KAPPA value of the classifier for the classification results was 0.688, representing a good performance. Two external independent validation sets were employed to test the efficacy of the classifier, and the final results corroborated that CS2 had better prognostic effects and similar differences in other aspects. To make our study reproducible and truly applicable to clinical research, we uploaded our packaged classifier to GitHub. Researchers can accurately classify patients with only RNA-seq data and clinical information, which might provide a reference for future clinical practice.

From the perspective of patient survival analysis alone, CS2 patients had longer overall survival time, with similar results in both the training and validation sets. However, predicting patient prognostic effects was only one function of the classifier. This study also explored other aspects such as the differences between the two subtypes in drug response, immune microenvironment, and biology. Even if CS1 had a higher probability of poor prognosis, we still hoped to present the various differences between the two subtypes at the genetic level to find suitable treatment strategies for each and apply them to clinical practice.

As many studies have shown that MPS played a crucial role in drug delivery, a higher proportion of MPS can lead to the dissolution of drug carriers, thereby enhancing patient resistance to drugs. Therefore, this study focused on the performance of the two subtypes in the GDSC database. CS2 presented a more sensitive response to Paclitaxel, 5-fluorouracil, Erlotinib, and Pazopanib, indicating that they may have better efficacy in clinical trials for patients in CS2. Paclitaxel therapy produced promising results in HNSCC patients and was successfully combined with cisplatin, carboplatin and/or etoposide in patients with advanced HNSCC (Spencer and Faulds 1994; Hitt et al. 2012). Given paclitaxel included Abraxane, a nanoformulation, in the GDSC, differences in patient drug response can indirectly reflect the impact of MPS on nanocarriers. 5-Fluorouracil was a major antimetabolite drug that was widely used in patients with advanced and recurrent HNSCC, which often appeared in clinical treatment (Yasumatsu et al. 2009; Lima et al. 2021; Psyrri et al. 2017). The EGFR signaling pathway contributed to the development and progression of HNSCC, and the EGFR-targeted drug erlotinib had shown good efficacy in the treatment of HNSCC (Gross et al. 2014; Stanam et al. 2015; Allen et al. 2015; Xu et al. 2017). For patients with recurrent or metastatic HNSCC, 800 mg/day of the CSF1R-targeted drug Pazopanib oral suspension in combination with standard weekly cetuximab had observed encouraging preliminary anti-tumor activity (Dincer et al. 2019; Adkins et al. 2018). From the analysis of the above drugs, which are clinically commonly used, targeting EFGR, CSF1R, and containing nanocarriers, we concluded that CS2 was more sensitive to these small molecular compounds. In other words, these drugs may be preferred when patients were identified as CS2 type. Furthermore, our study found CS2 patients who treated radiation therapy were more sensitive to 5-fluorouracil and Pazopanib compared to those without radiation therapy, indicating a combination strategy can be an effective way to improve the status of patients. Since the subtypes in this study were clustered based on multi-omics data related to MPS, it was reasonable to believe that MPS had a certain impact on the efficacy of these drugs.

CS2 had higher scores in most B cells and T cells in all three immune infiltration analyses, indicating a higher proportion of cells and immune effects. Overall, the immune system of CS2 can better inhibit HNSCC tumors, resulting in a better prognosis and drug response for patients. The biological functions analysis of CS2 illustrated that most pathways were enriched in metabolic pathways, which might be related to MPS. Further metabolic analysis also indicated that CS2 had better performance in overall metabolic, fatty acid metabolism, and cholesterol metabolism, reflecting CS2’s strong biological feature of xenobiotic metabolism. This phenomenon can be seen in some clues from immune infiltration analysis, which CS2 had lower proportions of MPS such as macrophages and DCs. Previous literature had shown that metabolism was a crucial underpinning of MPS (Davies et al. 2019), and its functional heterogeneity required diverse metabolism. MPS such as macrophages must induce or inhibit metabolic pathways to find, ingest, and digest apoptotic cells (Trzeciak et al. 2021). Metabolic interactions occurred when these cells came into contact with other cells, such as the stress-related metabolism produced by the interaction between NKT cells and MPS (Cortesi et al. 2018). In addition, MPS was also involved in the process of endogenous lipid oxidation-induced metabolism (Gioia and Zanoni 2021). In other aspects, we also compared the mutation differences between the two subtypes, in which CS2 had higher TMB and FGA, representing patients in CS2 were more likely to benefit from immunotherapy.

Although CS1 was considered to have a worse prognosis, the analysis of immune checkpoint inhibitors (ICIs) depicted higher expression of CS1 in many targets such as CD47, ABCA1, and MFGE8, which might be potential therapeutic targets. These targets had recently been shown to be associated with efferocytosis. In cancer treatment, CD47, as the classic “don’t eat me” signal of efferocytosis, was often overexpressed in apoptotic cell clearance defects and pathological blockade of CD47-SIRP1 interaction (Mehrotra and Ravichandran 2022). ABCA1 expression defects can lead to decreased efferocytosis in disease, and it seemed to have a beneficial effect on efferocytosis and anti-inflammatory signal transduction in vivo (Tang and Oram 2009; Wang and Oram 2005; Wang and Oram 2007; Joseph et al. 2002). Inflammatory-induced TLR signaling can further inhibit the expression of ligands such as MFGE8, thereby inhibiting increased efferocytosis and furthering disease pathology (Komura et al. 2009). Further enrichment analysis demonstrated that CS1 exhibited specificity in coagulation function, ECM, and JAK-STAT signaling pathway. In HNSCC patients, high platelet count and low tumor stroma ratio were closely associated with increased metastasis and poor prognosis. STAT3 and STAT5 were expressed and activated in HNSCC, contributing to cell survival and proliferation. In HNSCC, STAT can be activated by various signaling pathways, including EGFR, α7 nicotinic receptor, interleukin receptor, and erythropoietin receptor pathways (Lai and Johnson 2010). Due to the characteristic gene enrichment of CS1 in this target, future research can apply JAK inhibitors to clinical treatment when patients were identified as CS1 (Hwang et al. 2021).

## Conclusion

In this study, we identified two HNSCC subtypes and explored their biological difference. Based on the specific genes from the two subtypes, we established the relevant classifier, contributing to implementing precise treatment for HNSCC patients. Combined with other clinical characteristics, we hope our study can guide therapeutic strategies and offer some clues between MPS and HNSCC.

### Limitations

The study also has some limitations. In drug response analysis, the reference database only has response data of single drug action so we are unable to conduct sensitivity testing on the combined small molecule drug regimen. We look forward to more researchers delving deeper into drug response for cell lines to provide more data for future research.

## Supplementary Information

Below is the link to the electronic supplementary material.

Supplementary file1 (TIF 2572 KB)

Supplementary file2 (TIF 5434 KB)

Supplementary file3 (TIF 7352 KB)

Supplementary file4 (TIF 8416 KB)

Supplementary file5 (TIF 10364 KB)

Supplementary file6 (TIF 10924 KB)

Supplementary file7 (TIF 6618 KB)

Supplementary file8 (TIF 6197 KB)

Supplementary file9 (TIF 7512 KB)

Supplementary file10 (TIF 8082 KB)

Supplementary file11 (TIF 7832 KB)

Supplementary file12 (TIF 14860 KB)

Supplementary file13 (TIF 7000 KB)

Supplementary file14 (DOCX 18 KB)

Supplementary file15 (CSV 3 KB)

Supplementary file16 (XLSX 14 KB)

Supplementary file17 (XLSX 68 KB)

## Data Availability

The datasets generated and analyzed during the current study are available in the Cancer Genome Atlas (TCGA) repository (https://portal.gdc.cancer.gov/) and GEO database (https://www.ncbi.nlm.nih.gov/geo).
