# Supplementary figures and images for: Mononuclear phagocyte system-related multi-omics features yield head and neck squamous cell carcinoma subtypes with distinct overall survival, drug, and immunotherapy responses
Source: J Cancer Res Clin Oncol. 2024 Jan 27;150(2):37. doi: 10.1007/s00432-023-05512-5 (PMC10817853; doi:10.1007/s00432-023-05512-5)

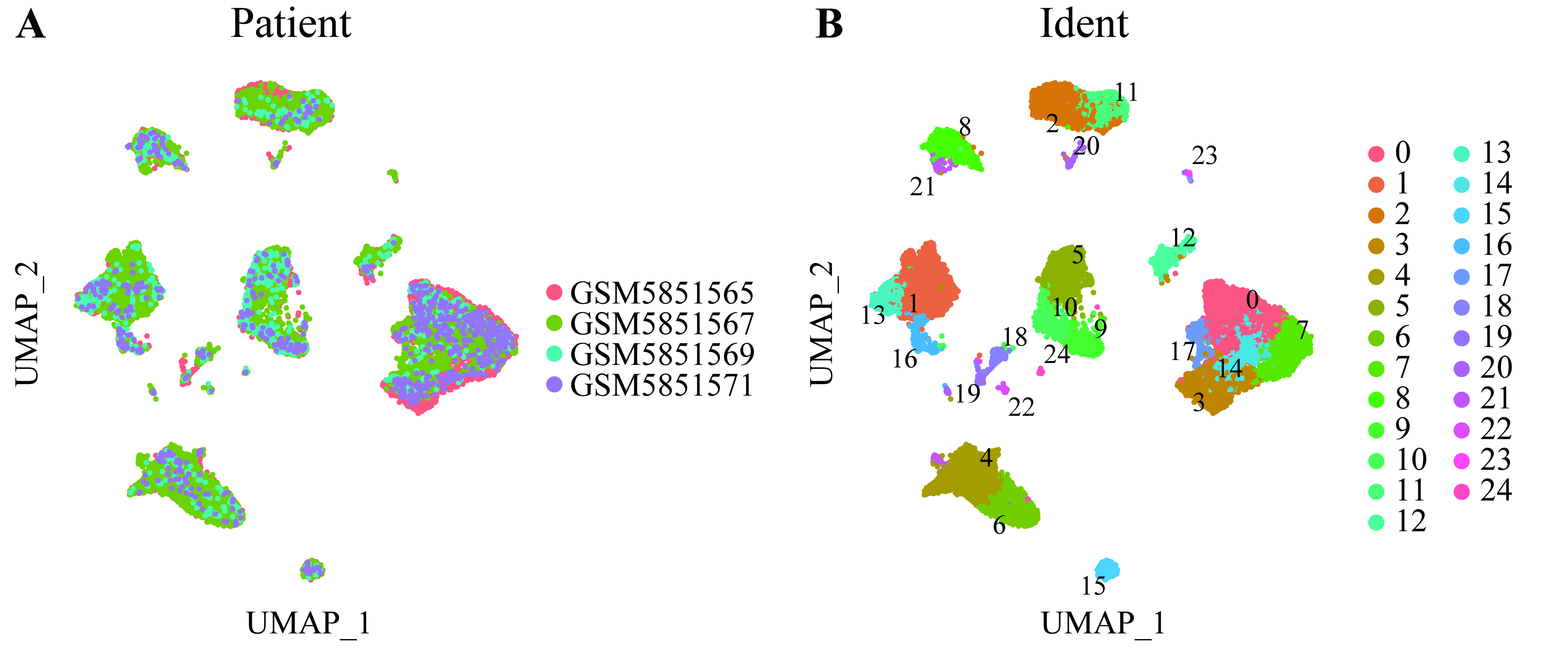

Supplement: Supplementary file 1 — Supplementary file1 (TIF 2572 KB) [file 432_2023_5512_MOESM1_ESM.tif]

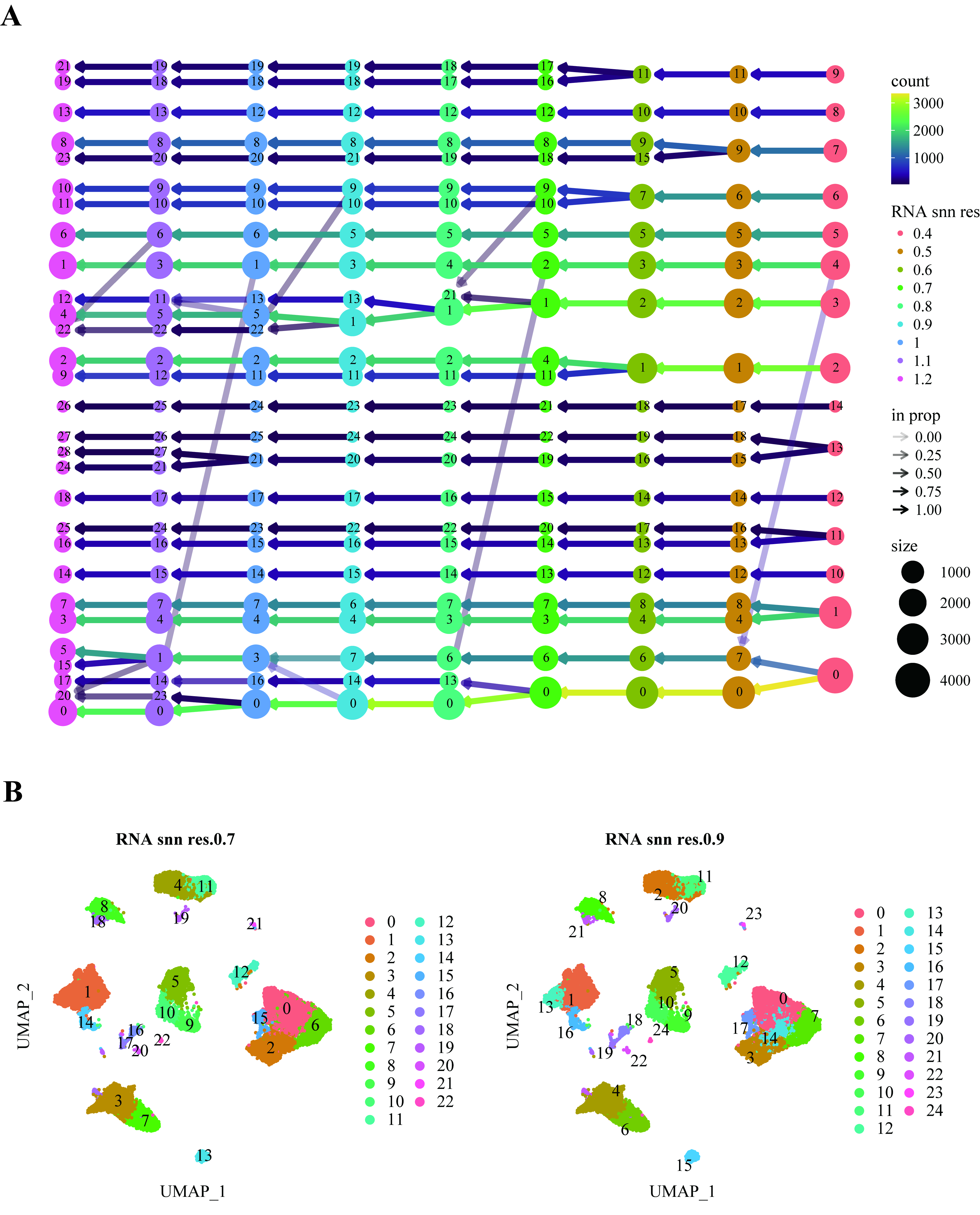

Supplement: Supplementary file 2 — Supplementary file2 (TIF 5434 KB) [file 432_2023_5512_MOESM2_ESM.tif]

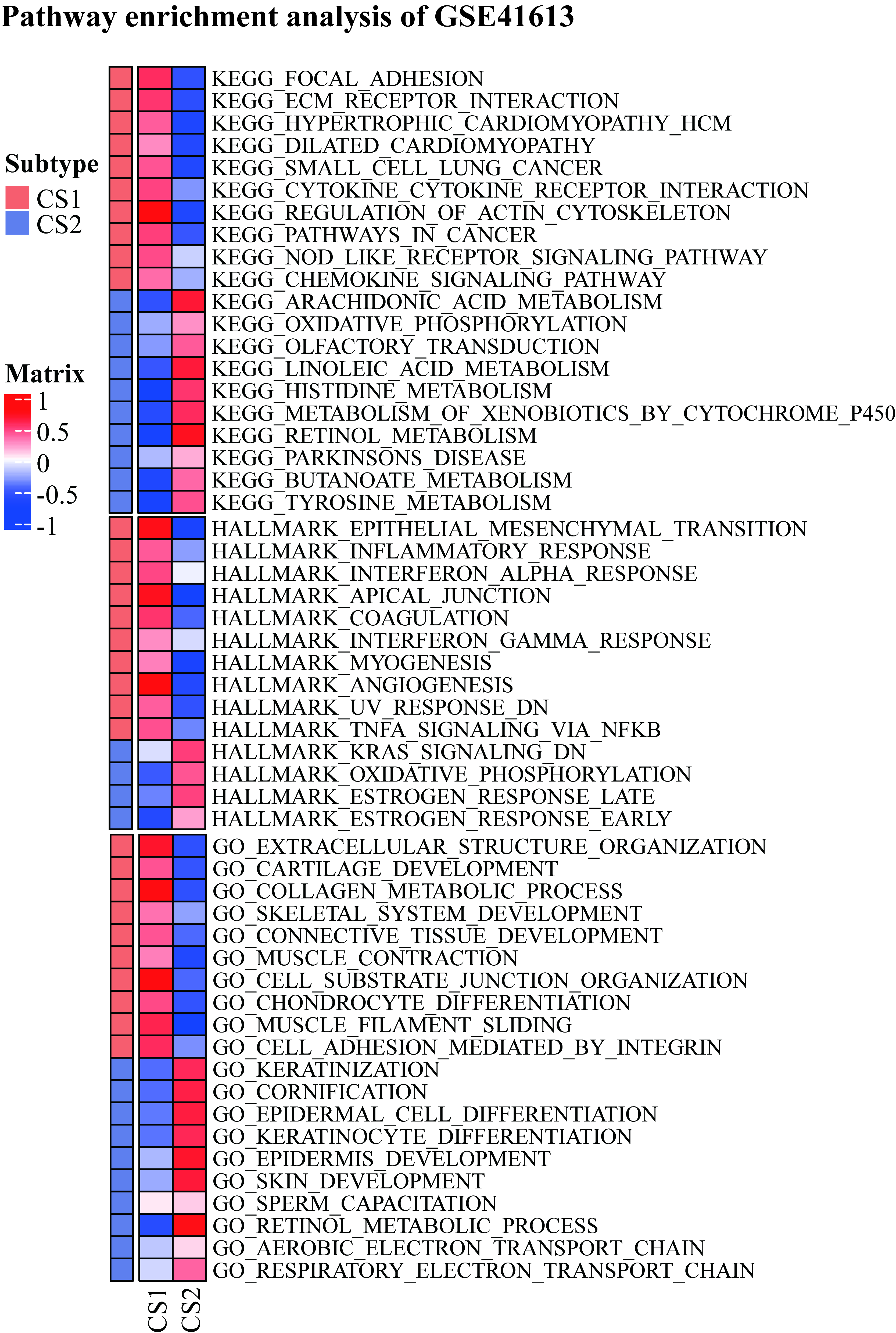

Supplement: Supplementary file 3 — Supplementary file3 (TIF 7352 KB) [file 432_2023_5512_MOESM3_ESM.tif]

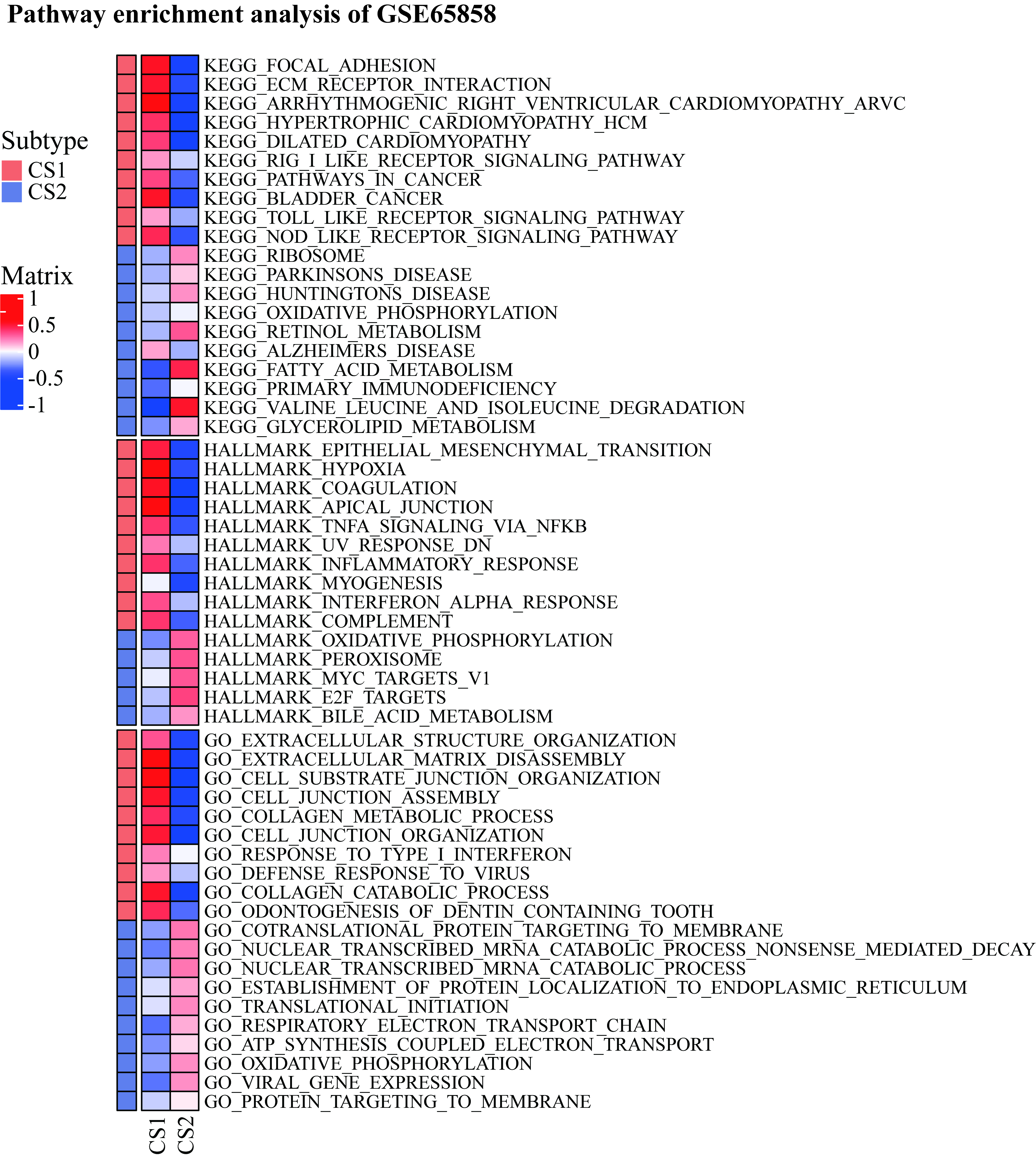

Supplement: Supplementary file 4 — Supplementary file4 (TIF 8416 KB) [file 432_2023_5512_MOESM4_ESM.tif]

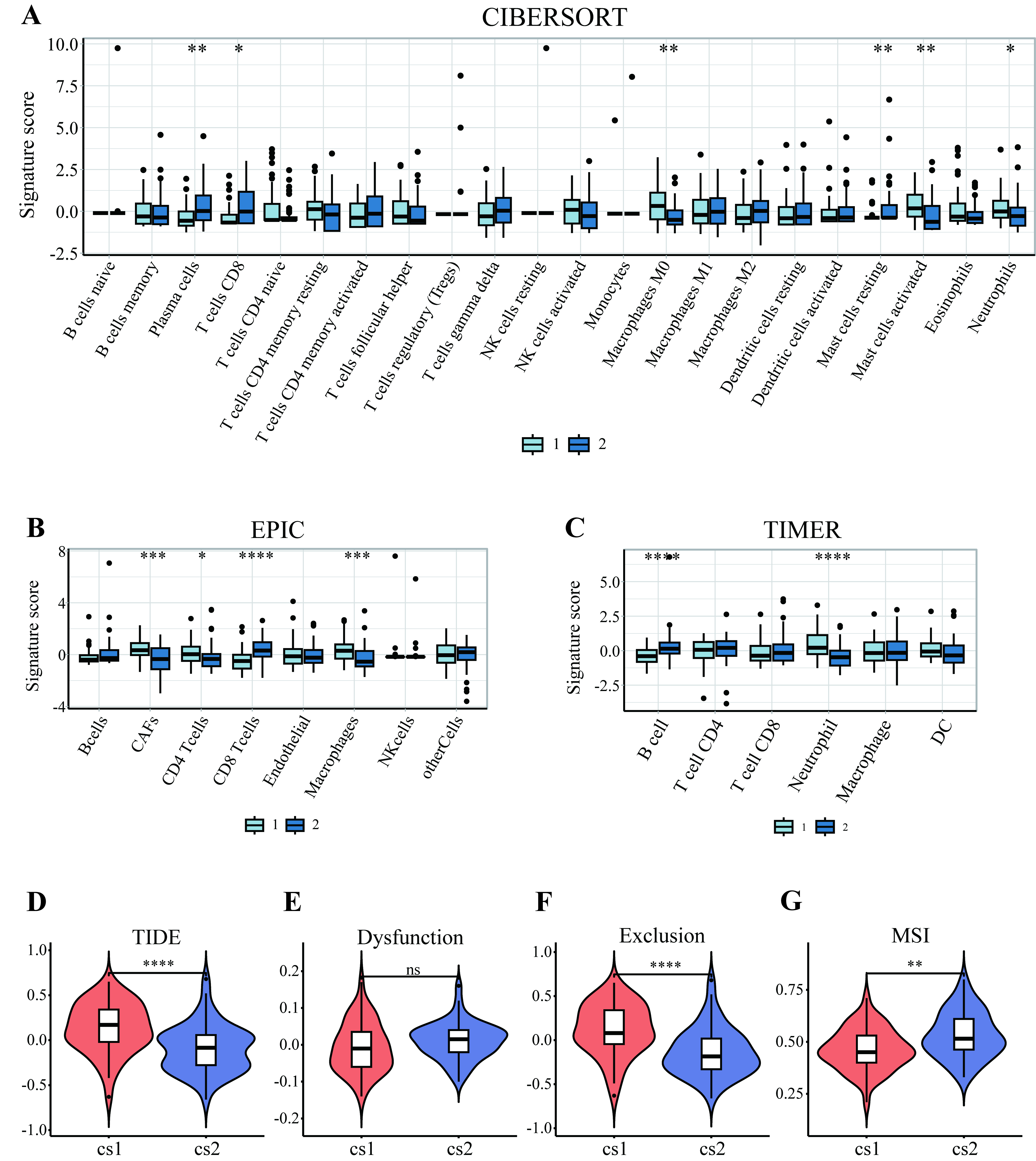

Supplement: Supplementary file 7 — Supplementary file7 (TIF 6618 KB) [file 432_2023_5512_MOESM7_ESM.tif]

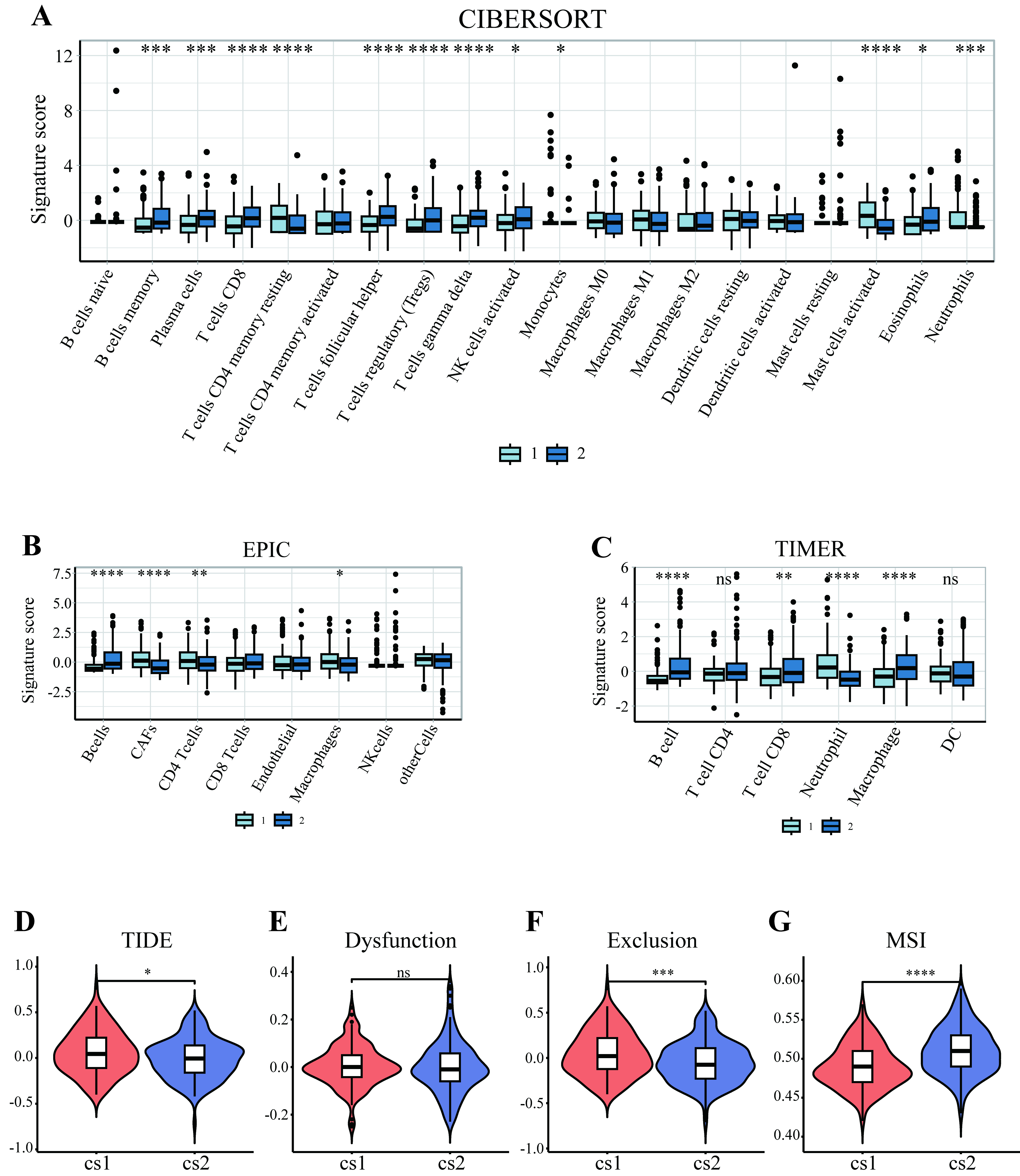

Supplement: Supplementary file 8 — Supplementary file8 (TIF 6197 KB) [file 432_2023_5512_MOESM8_ESM.tif]

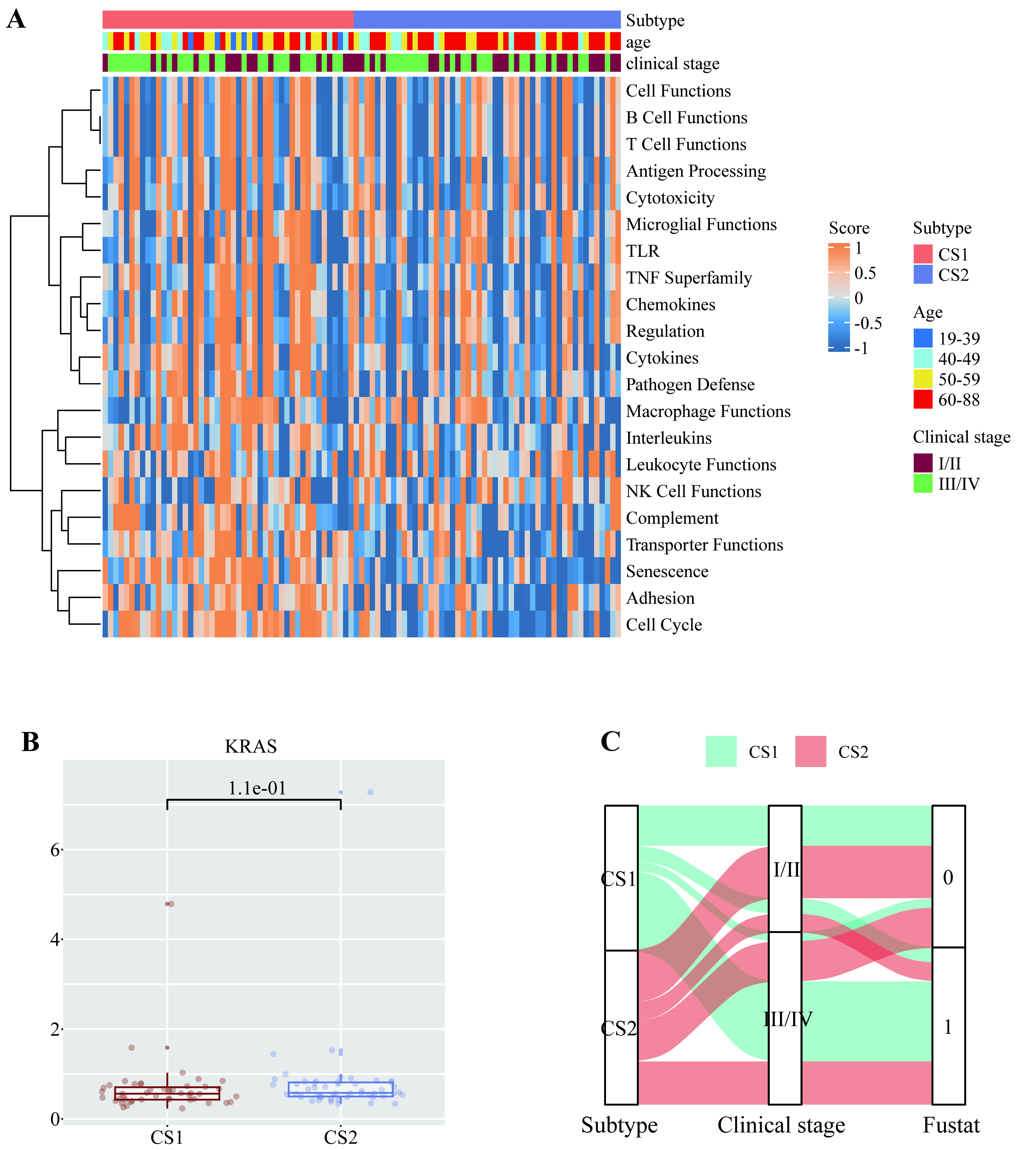

Supplement: Supplementary file 11 — Supplementary file11 (TIF 7832 KB) [file 432_2023_5512_MOESM11_ESM.tif]

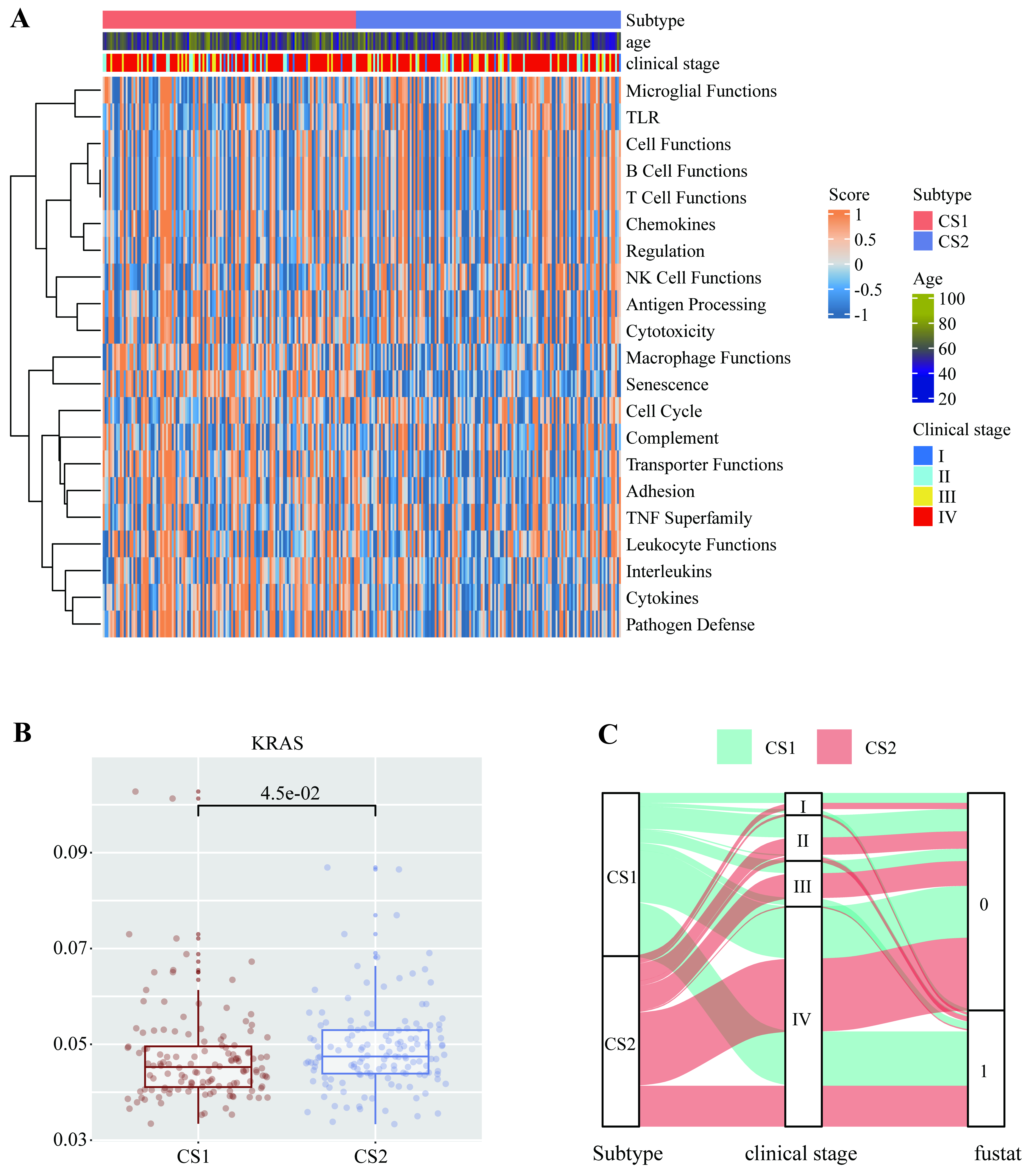

Supplement: Supplementary file 12 — Supplementary file12 (TIF 14860 KB) [file 432_2023_5512_MOESM12_ESM.tif]

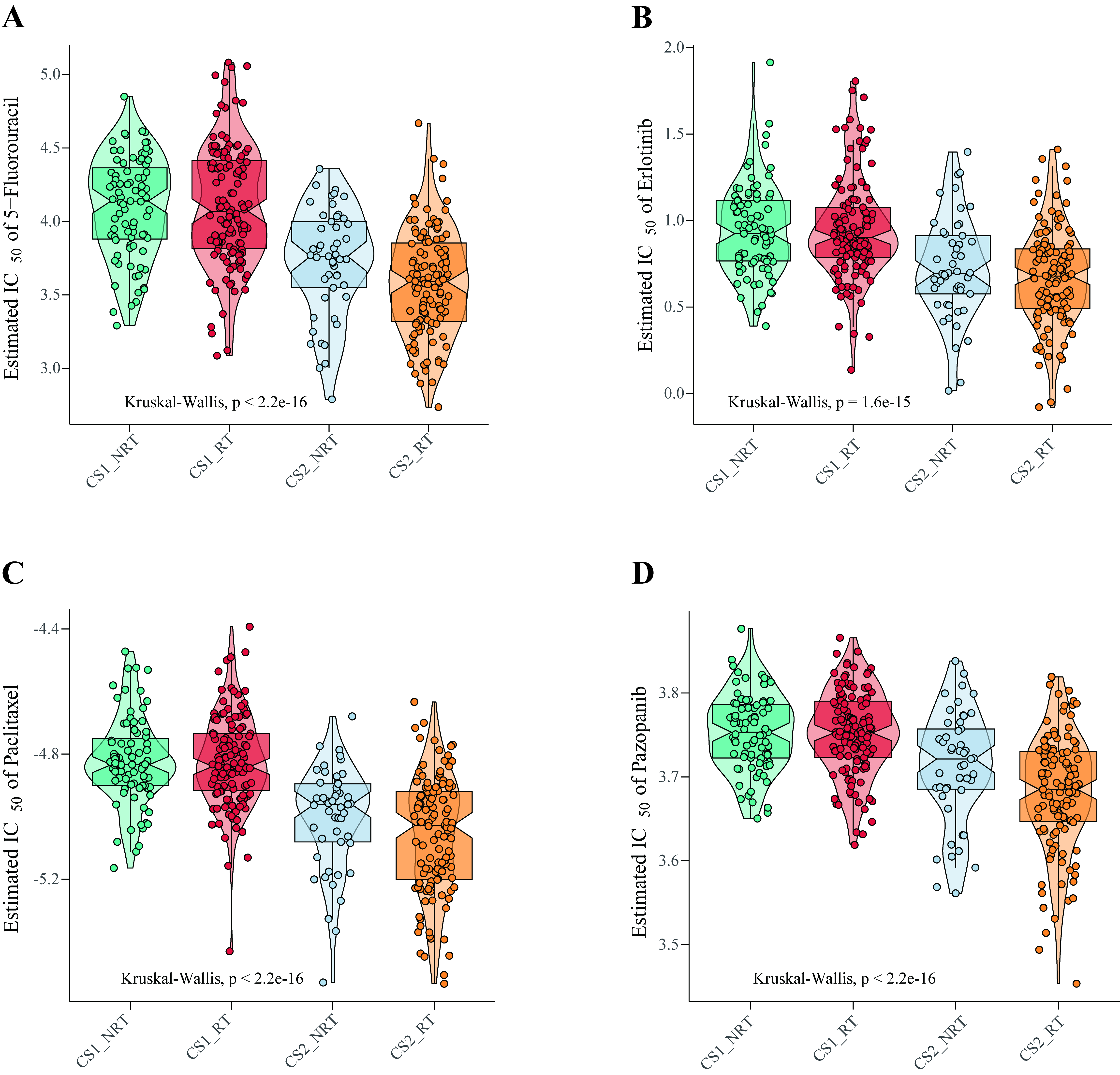

Supplement: Supplementary file 13 — Supplementary file13 (TIF 7000 KB) [file 432_2023_5512_MOESM13_ESM.tif]
