# Supplementary material for: Mononuclear phagocyte system-related multi-omics features yield head and neck squamous cell carcinoma subtypes with distinct overall survival, drug, and immunotherapy responses
Source: J Cancer Res Clin Oncol. 2024 Jan 27;150(2):37. doi: 10.1007/s00432-023-05512-5 (PMC10817853; doi:10.1007/s00432-023-05512-5)
Supplement: Supplementary file 14 — Supplementary file14 (DOCX 18 KB) [file 432_2023_5512_MOESM14_ESM.docx]

**Figure S1.** The process of single-cell annotation. **(A)** The distribution of cells after removing batch effects from different samples. **(B)** The number of clusters at a resolution of 0.9.

**Figure S2.** The process of selecting a resolution. **(A)** The “Clustree” algorithm presented the selection of cluster numbers at different resolutions. **(B)** The comparison of different resolutions, 0.7 and 0.9.

**Figure S3.** Functional analysis of two subtypes in GSE41613.

**Figure S4.** Functional analysis of two subtypes in GSE65868.

**Figure S5.** Metabolism analysis of two subtypes in GSE41613. **(A)** The overall metabolism analysis between two subtypes in GSE41613. **(B)** The fatty acid metabolism analysis between two subtypes in GSE41613. **(C)** The cholesterol metabolism analysis between two subtypes in GSE41613.

**Figure S6.** Metabolism analysis of two subtypes in GSE65868. **(A)** The overall metabolism analysis between two subtypes in GSE65868. **(B)** The fatty acid metabolism analysis between two subtypes in GSE65868. **(C)** The cholesterol metabolism analysis between two subtypes in GSE65868.

**Figure S7.** The comparison of two subtypes’ immune cell infiltration in GSE41613. **(A)** Differences in 22 immune cells between two subtypes by algorithm CIBERSORT. **(B)** Differences in eight immune cells between two subtypes by algorithm EPIC. **(C)** Differences in six immune cells between two subtypes by algorithm TIMER. **(D)** The TIDE score of two subtypes. **(E)** The Dysfunction score of two subtypes. **(F)** The Exclusion score of two subtypes. **(G)** The MSI score of two subtypes.

**Figure S8.** The comparison of two subtypes’ immune cell infiltration in GSE65858. **(A)** Differences in 22 immune cells between two subtypes by algorithm CIBERSORT. **(B)** Differences in eight immune cells between two subtypes by algorithm EPIC. **(C)** Differences in six immune cells between two subtypes by algorithm TIMER. **(D)** The TIDE score of two subtypes. **(E)** The Dysfunction score of two subtypes. **(F)** The Exclusion score of two subtypes. **(G)** The MSI score of two subtypes.

**Figure S9.** MPS-related analysis in GSE41613. **(A)** The specific proportion of macrophages between two subtypes. **(B)** The specific proportion of DCs between two subtypes. **(C)** The specific proportion of monocytes between two subtypes. **(D-K)** The expression level of TLR8, CSF1R, EGFR, CXCR4, ABCA1, MFGE8, CD47, and CX3CL1, between two subtypes.

**Figure S10.** MPS-related analysis in GSE65858. **(A)** The specific proportion of macrophages between two subtypes. **(B)** The specific proportion of DCs between two subtypes. **(C)** The specific proportion of monocytes between two subtypes. **(D-K)** The expression level of TLR8, CSF1R, EGFR, CXCR4, ABCA1, MFGE8, CD47, and CX3CL1, between two subtypes.

**Figure S11.** Additional functional analysis of two subtypes in GSE41613. **(A)** The difference of biological function between two subtypes. **(B)** The expression level of KRAS between two subtypes.  **(C)** Consistency between subtypes and other clinical phenotypes.

**Figure S12.** Additional functional analysis of two subtypes in GSE65858. **(A)** The difference of biological function between two subtypes. **(B)** The expression level of KRAS between two subtypes.  **(C)** Consistency between subtypes and other clinical phenotypes.

**Figure S13.** The comparison of four subtypes’ drug sensitivity. **(A)** The estimated IC50 of 5-fluorouracil between four subtypes. **(B)** The estimated IC50 of Erlotinib between four subtypes. **(C)** The estimated IC50 of Paclitaxel between four subtypes. **(D)** The estimated IC50 of Pazopanib between four subtypes. (CS1_NRT, CS1 patients with no radiotherapy, CS1_RT, CS1 patients with radiotherapy, CS2_NRT, CS2 patients with no radiotherapy, CS2_RT, CS2 patients with radiotherapy)

**In all figures, ns/empty space, no significance, * p < 0.05, ** p < 0.01, *** p < 0.001, and **** p < 0.0001**

**Table S1.** The results of Enrichr.

**Table S2.** The list of genes expressed in CS1 and CS2 used to construct the classifier.

**Table S3.** The list of the MPS-related biomarkers.
